# Immunity-based Ebola optimization search algorithm for minimization of feature extraction with reduction in digital mammography using CNN models

**DOI:** 10.1038/s41598-022-22933-3

**Published:** 2022-10-26

**Authors:** Olaide N. Oyelade, Absalom E. Ezugwu

**Affiliations:** School of Mathematics, Statistics, and Computer Science, University of KwaZulu-Natal, King Edward Avenue, Pietermaritzburg Campus, Pietermaritzburg, 3201 KwaZulu-Natal South Africa

**Keywords:** Cancer, Mathematics and computing

## Abstract

Feature classification in digital medical images like mammography presents an optimization problem which researchers often neglect. The use of a convolutional neural network (CNN) in feature extraction and classification has been widely reported in the literature to have achieved outstanding performance and acceptance in the disease detection procedure. However, little emphasis is placed on ensuring that only discriminant features extracted by the convolutional operations are passed on to the classifier, to avoid bottlenecking the classification operation. Unfortunately, since this has been left unaddressed, a subtle performance impairment has resulted from this omission. Therefore, this study is devoted to addressing these drawbacks using a metaheuristic algorithm to optimize the number of features extracted by the CNN, so that suggestive features are applied for the classification process. To achieve this, a new variant of the Ebola-based optimization algorithm is proposed, based on the population immunity concept and the use of a chaos mapping initialization strategy. The resulting algorithm, called the immunity-based Ebola optimization search algorithm (IEOSA), is applied to the optimization problem addressed in the study. The optimized features represent the output from the IEOSA, which receives the noisy and unfiltered detected features from the convolutional process as input. An exhaustive evaluation of the IEOSA was carried out using classical and IEEE CEC benchmarked functions. A comparative analysis of the performance of IEOSA is presented, with some recent optimization algorithms. The experimental result showed that IEOSA performed well on all the tested benchmark functions. Furthermore, IEOSA was then applied to solve the feature enhancement and selection problem in CNN for better prediction of breast cancer in digital mammography. The classification accuracy returned by the IEOSA method showed that the new approach improved the classification process on detected features when using CNN models.

## Introduction

The application of deep learning (DL) architectures to text-based medical records and image analysis has revolutionized healthcare service and delivery. Computational solutions driven by DL models have proven successful in several aspects, ranging from detection, image analysis, classification, diagnosis, synthetization of medical images, and record management^1–9^, against the traditional computerized diagnostic systems (CADs)^10–13^. Convolutional neural networks (CNN) and recurrent neural networks (RNN) are examples of DL models widely used for these tasks. Image preprocessing methods often precede the process flow of CNN, followed by the convolutional-pooling operation, which detects suggestive features that lead to the classification of the image as normal or abnormal. The performance of the feature extraction process, shadowed by convolutional-pooling operations, largely depends on a well-architected CNN model. The need for improved performance of these models is motivated by classification accuracy, which supports the acceptability of the deployment of the model for domain application. As a result, several techniques have been proposed to address drawbacks, such as overfitting, and inefficient parameter and hyperparameter combination or selection, which are associated with CN design. For instance, choice image preprocessing, data augmentation^14^, optimization of weights and hyperparameters using metaheuristic algorithms^2^, auto-design of CNN^15^, and many more, have shown to be promising techniques in addressing those drawbacks. Deserving further mention is the use of metaheuristic algorithms which have played multi-functional roles in hyping the performance and use of DL models. Such roles include parametric modeling^16^, architectural reconfiguration, adapting natural phenomena to resolve drawbacks and designing DL models, influencing training so that peak performance is achieved faster, finding neo-optimal solutions to classification problems at a reduced computational cost^17^, extraction of insightful patterns from huge data representing medical images or big data^18^, and their performance enhancements with classification issues. Their cost-effective measures addressing these challenges have advantaged and endeared metaheuristic algorithms to researchers in furtherance of the performance of DL.

Optimization algorithms, also known as metaheuristic algorithms, have proffered solutions not only to complex problems associated with DL but to various research domains demonstrating difficulty in finding the best solutions, including engineering and science. They have mostly been inspired by natural, physical, and chemical processes, which inherently possess patterns for computational solutions to those complex problems. These algorithms are categorized into evolutionary-based, drawn from the law of natural selection and evolutionary computing; swarm-based, inspired by nomadic and social interaction among animals; physics-based, cued from laws represented in physics; human-based, motivated by intelligent behavioral patterns among humans; biology-based, guided by interactivity among microorganisms; system-based, derived from well-represented systems in nature; math-based, drawn from standard mathematics models; and music-based, following the harmonious rhythm of musical notes. In our recent studies, we have proposed a novel biology-based metaheuristic algorithm, Ebola optimization search algorithm (EOSA)^19,20^, which demonstrated a fast convergence rate and high accuracy when applied to solve various problems relating to DLs.

Interestingly, studies have shown that variants of optimization algorithms have often outperformed the original method, so that problems addressed using such algorithms are further improved through a proper search for better solutions^21^. These variants are often motivated by observing the weakness of the underlying or base algorithm, leading to further derivation of new concepts and operations associated with the domain of inspiration of the algorithm, and challenging new problems, known with real-life problems, which the algorithm has failed to fully address, as well as the need for diversifying the search process of the algorithm. Concepts like balancing exploration and exploitation phases, hybridization with another well-performing optimization algorithm, widening and maturing of the search space, parametric control, and convergence rate have shaped the development of these new variants. Careful consideration of the novel EOSA metaheuristic algorithm performance, with respect to the domain of application and its source of inspiration, has revealed that the algorithm can be further developed to address upcoming problems in the same domain of DL. One of these problems is optimizing the number of features passed on from the convolutional-pooling layers to the classification function, so that only discriminant features are selected for the image classification task in CNN. This rarely harnessed area of research presents an interesting study on improving classification accuracy through improving the quality and quantity of features obtained during the feature extraction stage of the CNN operation.

The task of feature extraction using DL models has been presented as a black box which has prevented attempts at improving the models at this stage^22^. Much has been reported on image pre-processing, choice of classifier, hyperparameter selection, architectural design, and weight optimization as performance improvements in DL models. However, little or no attention is given to increasing the need to scale down the extracted features resulting from the convolutional-pooling layers. The challenge with using such features without further filtering is a bottlenecked classifier which is overwhelmed with relevant and non-relevant features with noise capable of maligning probability maps. Although attempts have been made to address this challenge, the problem remains relevant with the most celebrated state-of-the-art DL classification models for medical images. Even the few studies which have considered this challenge have only applied methods using peak-inflection point, discrete cosine transform (DCT), discrete wavelet transform (DWT), singular value decomposition (SVD), staked CNN architectures, dual-stream deep architecture, dimensionality reduction like principal component analysis (PCA), feature fusion which improves features resulting from feature extraction using deep convolutional neural network (DCNN), and CNN-based auto features extraction (CNN-AFE). For example, Sahlol et al.^23^ applied Salp Swarm Algorithm (SESSA) to improve the selection of preferred detected features leading to the classification of White Blood Cell (WBC) Leukaemia in samples; Fatani et al.^24^ used the traditional method of CNN for extracting features, and then applied Aquila Optimizer (AQU) for further feature selection; in^25^, the Grasshopper Optimization Algorithm and the Crow Search Algorithm were hybridized to address the challenge of feature selection leading to classification using MLP. Whale Optimization Algorithm (WOA), which was hybridized with Flower Pollination Algorithm (FPA), was investigated in^26^ for feature selection for email. Similarly, a binary variant of the Symbiotic Organisms Search (SOS) Algorithm has also been applied to feature selection tasks in the detection of spam in emails^27^, and other feature selection problems^28^. In^29^, the Advanced Squirrel Search Optimization Algorithm (ASSOA) was used to scale down the number of detected features from CNN (ResNet-50 specifically). Farmland Fertility Algorithm (FFA) has been used to optimize the task of feature selection in handling the performance of intrusion detection systems^30^. The use of the metaheuristic algorithm approach presents viable solutions to the challenge of enhancement of feature extraction using CNN.

To address the limitation highlighted in the previous paragraph, this study proposes a new variant of EOSA adaptable for solving the problem based on suitable features added to its process of mutating solutions. Recall that Ebola virus disease (EVD) is a severe and frequently lethal disease caused by the Ebola virus (EBOV)^31^. Those outbreaks were associated with a massive fruit bat migration through the region^32^. Cardinal among the added features to EOSA is the concept of an infected-susceptible immunity factor (ISMF), which allows for an intelligible mutation of individuals presented as either infected or those who remain in the susceptible group in the search space. This is motivated by studies which have shown that robust adaptive immunity is activated during the acute stage of the Ebola virus so that competition exists between EBOV proliferation and the ability of the human host to mount an effective and regulated anti-EBOV immune response^31,33^. Weaving this with other important features into EOSA presents an improved optimizer useful for restructuring CNN architecture so that outlier, discriminant, and suggestive features crowded by noisy features in buffered extracted features are filtered out to get the optimal performance of predictive models for classification tasks. This process not only improves performance, such as good generalization and classification accuracy, but also makes attainment of best performance faster, eliminates redundancy and model overfitting, and improves precision, as observed in^34^. The following describes the contribution of this study to the advancement of research: presents a new structure of CNN interleaved with a feature optimization mechanism. The optimization mechanism is designed from an infected-susceptible immunity factor (ISMF) and improved search process, resulting in a new variant of EOSA. Extracted features from the CNN architecture are then scaled down for performance improvement. An exhaustive evaluation of the new variant IEOSA was presented using classical benchmarked functions and then applied to solve the problem of enhancement of features in CNN for better prediction of breast cancer in digital mammography.

The following describes the contribution of the study:

- A new variant algorithm based on population immunity to virus spread is proposed for EOSA.
- A chaotic map initialization strategy is applied to investigate the performance of the resulting immunity-based EOSA.
- A CNN architecture is designed and trained to extract features from digital medical images.
- Features extracted are then optimally selected for classification purposes, using the new variant of EOSA.
- An exhaustive comparative analysis is performed on IEOSA, EOSA and other recent state-of-the-art metaheuristics algorithms.

The remaining sections in the paper are as follows: In “Related works” section, relevant studies on biology-based optimization and attempts made on feature extraction filters are discussed. “Methodology” section presents the methodology of the study, while “Experimentation setup” section provides readers with datasets and the configuration of the computational environment. The study’s experimental results and discussion are presented in “Results and discussion” section. Finally, the concluding remarks and future research direction are presented in “Conclusion” section.

## Related works

The use of metaheuristic algorithms to support the selection of optimal features from detected features using CNN is an interesting field of research. In this section, we present a review of some selected studies in this aspect of research. First, a review of the Ebola virus and its associated disease is presented, followed by a recently proposed optimization algorithm, EOSA^20^, which we developed based on the propagation of the disease. Considering the biology-based inspiration of EOSA, we review some related biology-inspired metaheuristics algorithms and their variants to present readers with trends in the design and advancement of biology-based optimization algorithms and their variants. This is necessary to support the idea presented in this study which aims to present a new variant of EOSA to address a problem in image feature optimization. Finally, we conclude this section with studies which have improved the feature extraction of digital images in deep learning, using methods such as metaheuristic algorithms.

### Ebola virus and EOSA

The Ebola virus (EBOV) often results in Ebola disease, which is reported to be fatal and spreads proportionately to cover susceptible populations. The propagation strategy of the virus remains a challenge to health managers and has overwhelmed hospital infrastructure in places where this is known to be fragile. The virus is transmitted by direct, typically non-aerosol, human-to-human contact or contact with infected tissues, bodily fluids, or contaminated fomites. Public health officials generally consider disease transmission of infectious agents to fall into three categories—contact transmission (direct and indirect), droplet transmission, and airborne transmission. The spread of the disease stems from an infested host and pathogens (e.g. via bats) carrying the virus and then spreading to the human population. One entry into the human population has often propagated the disease among the human population, so an endemic has been reported. The immunity-lowering nature of the disease has been well researched, with studies showing that the virus first attacks important organs, degenerating to macrophages and dendritic immune cells forming the human immune system^35^. This activity notwithstanding, it has also been discovered that the human immune system does not just remain defenceless but also puts up a fight through an effective immune response at the same cellular level, so that levels of IgM and IgG are increased to fight off the disease^33^. This has enabled infected individuals to rise above the disease’s fatality and removed them from the line of potential wheels through which the virus spreads its infection among susceptible populations. This study investigates this phenomenon, among others, in developing a variant of the novel EOSA method.

The EOSA metaheuristics presented in our recent work^20^ leverage the nature of propagation of the Ebola virus and its associated disease to discover how exploration and exploitation phases of optimization might help to address some optimization problems in medicine. We improved the SIR model of Ebola to achieve a novel model named SEIR-HVQD. The model resulted from incorporating into the basic SIR (Susceptible, Infected, Recovered) model the concepts of Exposed (E), Hospitalized (H), Vaccinated (V), Quarantined (Q), and Death or Dead (D). The optimization process of the EOSA method was illustrated using a set of mathematical equations demonstrating the approach’s usefulness in solving NP-Hard problems, which are optimization problems in nature. Using a wide range of benchmark functions, we proved that the method remains valid and is competitive with some related state-of-the-art methods. In addition, the applicability of the EOSA method was investigated by addressing optimization problems known with fine-tuning of DL architectures. This became possible using an algorithm which follows the procedure listed here:

1. Initialization of individuals in the compartments Susceptible (S), Infected (I), Recovered (R), Dead (D), Vaccinated (V), Hospitalized (H), and Quarantined (Q) was achieved through the computation of the vector representation of the CNN architecture. Although all compartments were initialized to null except for S, this allows for the optimization process to achieve its aim.
2. Considering the necessity of an index case for propagation in the human population, we randomly generated an index case from the infected to aggress towards susceptible individuals.
3. The index case is set as the global best and current best to allow for searching for other solutions presenting more comparable outcomes over the index case.
4. The procedure is iteratively trained to allow for an exhaustive search and reconfigurations of individuals in the search space.
  - during each phase of these iterations, every infected and susceptible individual is updated based on the occurrence of infection, so that we can update their positions based on their displacement.
    i. Generate newly infected individuals.
    ii. Add the newly generated infected cases to the existing list of infected.
  - Compute the number of individuals to be added to H, D, R, V, and Q, using their respective rates based on the size of I.
  - Update S and I based on new infections.
  - Select the current best from infected individuals and compare it with the global best.
  - If the condition for termination is not satisfied, go back to step 4.
5. Return the best solution describing the combination of weights and bias suitable for solving the classification problem in the DL model whose vector solution has been optimized.

The above describes how the notion of the optimization process using metaheuristic algorithms has been drafted to address the challenge of optimizing the weights of CNN architectures. Interestingly, this solution has been widened to address the bigger challenge of selecting the best hyperparameters suitable for configuring a DL model that can address the same classification problem. Further to this, studies like^15^, which is one of our research outputs, have even advanced research using metaheuristic algorithms in automating the design and building of DL architectures so that the same classification problem is addressed. In this study, we further advance research in this direction by focusing on detected features rather than only the architecture, so that we minimize the number of features the classifier will have to sieve through in addressing the classification problem. As a result, we seek to improve the EOSA algorithm to suit it for the new dimension of the problem. Meanwhile, we first review how other related algorithms, such as EOSA, have been improved to address optimization problems for which they are effective.

### Bio-inspired and disease-based optimization algorithms

The biology-based optimization algorithms have always been motivated by the biological processes inherent in most biological creatures, organisms, and microorganisms. Several metaheuristic algorithms that mimic these biological processes have been shown to be effective in addressing real-life optimization problems, considering their nature-inspired process of sustaining their underlying biological creatures. We review some of these algorithms and their related variants.

The first set of algorithms considered here are those inspired by viruses and their propagation nature. The Coronavirus Optimization (CVO) metaheuristic, which is based on the spread of the coronavirus and how the virus infected susceptible cases through the infected cases, has been presented in^36^. Concentrating on the super-spreading nature of the virus, authors have considered reinfection probability, super-spreading rate, social distancing measures, and traveling rate as parameters in designing the algorithm. The study also factored in mitigating measures such as social distancing and isolation, the mortality rate, and recoveries, to dynamically adjust solutions during the training of the algorithm. Interestingly, considering the different variants of the virus, the study naturalized the algorithm’s performance so that a wider search space is supplied for finding optimal solutions. The resulting CVO has been used to solve the combinatorial problem of finding the best selection of hyperparameters required for building a DL model in dealing with time series forecasting. In a related study^37^, a variant of CVO is being reported with emphasis on motivating the design of the algorithm based on the SIR model. Selected benchmark functions were used to evaluate the performance of the proposed version of the CVO, so that the problem of discrete and continuous mathematical functions is addressed. In another study^38^, the concept of herd immunity has been proposed to advance the CVO to achieve Coronavirus Herd Immunity Optimization (CHIO). The concept of herd immunity considers a case where a significant number of the population attains a level of immunity so that the spread of the virus is walled. This concept was then built into CHIO so that only the susceptible, infected, and immune individuals are considered. Evaluation of CHIO was experimented with using some benchmarked functions, those of IEEE-CEC 2011, and some engineering problems.

A generic coverage of applying inspiration from behavioral patterns of viruses with respect to the optimization process, was demonstrated in^39^. The study proposed a new metaheuristics algorithm designed based on the virus’s diffusion and infection strategy, which they called Virus Colony Search (VCS). The model follows how a virus moves into the host before infesting it for deeper propagation. Leveraging these two natural aspects of the virus, the algorithm can demonstrate the ability to explore and exploit the environment in search of the best solution. Evaluation of the method was investigated using CEC2014 benchmark functions, as well as some other problems, and numerical and constrained engineering design. Another related work^40^ proposed a new variant of VCS based on the concept of multi-objective problems often associated with cloud service providers. The Multi-Objective Virus Colony Search (MOVCS) was then applied in a model to handle three objects: Service Level Agreements Violation (SLAV), energy consumption, and a number of Virtual Machine Migrations (VMM), where all require minimization. Similarly, Syah et al.^41^ considered the same perspective of multi-objectivity in proposing a variant of VCS through a hybridization process. In their work, Particle Swarm Optimization (PSO) and VCS were hybridized so that the former improves search at local and global levels and the latter addresses the remaining optimization process in solving both linear and non-linear constraints problems. In another related work, Berbaoui^42^ also investigated the approach of hybridization of VCS with fuzzy logic, so that a fuzzy process is applied to design the objective functions in solving multi-objective problems relating to voltage sag, power factor, and total harmonic distortion (THD). The fuzzification of the objective function allows for minimizing values returned by the fitness function.

In^43^, the authors presented another biology-based algorithm called Invasive Weed Optimization Algorithm (IWO). The method drew inspiration from the evolutionary approach of weeds to solve optimization problems. The population-based algorithm leverages the invasive nature of weeds into economically viable plants, non-conducive environments notwithstanding. The concepts of robustness, adaptation, and randomness have been considered in the design of the algorithm, which has now proved useful in solving some optimization problems through evaluation with benchmark multi-dimensional functions and tuning a robust controller engineering problem. Similarly, the study in^44^ proposed the use of chaos theory to improve IWO to obtain CIWO, so that chaotic logistic maps are applied to the standard deviation parameter. The resulting variant of IWO was used to solve the problem of selecting the PID controller parameters so that it is evaluated based on the convergence rate and accuracy. Also, the work of^45^ developed a new variant of IWO based on discrete populations to address the problem of time–cost trade-off (TCT) and discrete benchmark problems. The DIWO algorithm was also applied to solving the optimization problem of unmanned aerial vehicles (UAVs), which requires congruent multiple task handling. In^46^, a hybrid method is used to improve IWO at the exploration phase to achieve an expanded version of the Invasive Weed Optimization Algorithm (exIWO).

As the biology processes continue to inspire the computational solutions to automated real-life problems, authors in^47^ proposed a new algorithm based on the geographical locations of biological organisms named Biogeography-Based Optimization (BBO). Mathematical models were formulated based on this distribution pattern of the organism, so that the optimization process is simulated. The BBO algorithm is also motivated by the migration strategy existing among these organisms. The resulting process gave an optimization algorithm used to solve the problem of selecting sensors in maintaining aircraft and addressing high-dimension problems with multiple local optima. In related work, Lim et al.^48^ addressed the weakness of BBO by formulating a hybrid variant so that the mutation aspect of BBO is improved with the Tabu search algorithm. The new variant was adapted to solve the Quadratic Assignment Problem (QAP), with the further evaluation carried out on selected benchmark instances in QAPLIB. The work of^49^ considered addressing discrete problems using an enhanced version of BBO so that problems typical of Single Machine Total Weighted Tardiness Problem (SMTWTP) are handled. Authors in^50^ also solved similar problems by developing a variant of BBO by adapting it to a non-constrained parameter penalty function to widen exploration capability. This variant has helped address the reliability redundancy allocation problems of the series–parallel system associated with a nonlinear resource. Identifying the limitation of BBO in solving problems prone to change in function values, the study of^51^ incorporated momentum to migration and taxonomic form to mutation, resulting in a new variant. This addition of momentum and taxonomic mutation allows the algorithm to stabilize when function values change and shields some potential solutions from being mutated dramatically or barely. The mathematical model of BBO has been improved to solve the truss structures with the natural frequency constraints problem^52^. The resulting version of BBO is called the Enhanced Biogeography-Based Optimization (EBBO) method, which is focused on improving the migration and mutation operators of the original BBO.

Related to organisms’ cohabitation as an inspiring solution to computational problems is the Satin Bowerbird Optimization Algorithm (SBO), first reported in^53^. The mating process exhibited by the Satin Bowerbird motivated the design of SBO since the male’s approach towards the female specie suggests an interesting phenomenon. The novel metaheuristics method was hybridized with an adaptive neuro-fuzzy inference system (ANFIS) to estimate the required efforts to complete software development projects. Recently, Wangkhamhan^54^ improved SBO by infusing a chaotic theory that uses a chaotic map to support the search process, yielding acceptable global convergence. The new variant was named Adaptive Chaotic Satin Bowerbird Optimization Algorithm (AC-SBO). It resulted in an improved method with its exploration and exploitation phases, as revealed in some tests done on benchmark functions. Similarly, the work reported in^55^ addressed the widely studied weakness of SBO, which is the search process. In this study, the search process was improved to auto-set the parameters of the qubit, which in turn updates the positions of the Bloch sphere mimicked by Satin Bowerbirds as best solutions are sought. The enhanced algorithm is considered a quantum-inspired SBO with Bloch spherical search. Authors in^56^ used an encoding strategy, namely Complex-Valued Satin Bowerbird Optimization Algorithm (CSBO), to address the same search process limitation, which often impaired the process of getting the global best.

The earthworm is another biological organism which has inspired the design of metaheuristic algorithms. Studies on the soil burrowing nature of earthworms, which replenishes the soil, have revealed that such symbiotic behavior presents a solution to optimization problems^57^. The study also considered the reproduction pattern of earthworms to consolidate the algorithm design. The new algorithm, called the Earthworm Optimization Algorithm (EWA), follows the reproductive process of earthworms in two phases so that the weighted sum of all offspring produces earthworms for the next generation. The EWA solves the problem of holing-up in local optima by relying on evolutionary concepts involving crossover operators motivated by similar differential evolution (DE) operations and genetic algorithms (GA). In addition to the crossover operation, the Cauchy mutation (CM) method was adapted to the algorithm to improve performance, which was evaluated using high-dimensional benchmark functions. The problem of routing and clustering related to finding the right cluster was solved using an enhanced EWA method using fractional calculus^58^. Using the idea of fit factor, cluster heads are selected to provide a route to sink nodes using the Fractional Calculus Earthworm Optimization Algorithm (FEWA). In another related study, Salunkhe^59^ developed a hybrid of EWA, which utilizes Discrete Cosine Transform (DCT) and Structured Similarity Index (SSIM) for the selection process in combination with Water Wave Optimization (WWO). The new variant is called Water-Earth Worm Optimization (WEWO) algorithm. The authors in^60^ also proposed a hybrid version of EWA based on its source of crossover operations which is DE, to achieve enhanced differential evolution (EDE) and Earthworm Optimization Algorithm (EWA), namely (EEDE). The hybrid algorithm was adapted to smart homes to minimize peak-to-average ratio (PAR) and energy cost.

In^61^, Wildebeest Herd Optimization (WHO) algorithm is proposed for global optimization tasks. This method derives its inspiration from the forage searching approach of wildebeest, which often move from low concentrations of grasses to highly concentrated regions. In addition, the algorithm leverages the herd form of habitation and relocation strategy seen in wildebeest and implements their lookout ability for little-grazed regions to ward off starvation. The limited sighting ability of the animals was explored to design local search for the best solution, while the nomadic lifestyle provides for global exploration.

The Slime Mould Algorithm (SMA) has been proposed in^62^ and described as a stochastic optimizer. The mathematical model was used to demonstrate the oscillation pattern of the slime mould so that waves generated by the positive and negative output of the slime mould are modeled. This was inspired by using the oscillation pattern for food search to maximize the whole search process. The design follows this oscillation to achieve both exploration and exploitation phases. The new algorithm was applied to address some classical engineering constrained problems. The unbalanced exploration and exploitation of SMA were addressed in a new variant using spiral search, chaotic theory, and parameter adaptive control methods^63^. The new variant, MSMA, successfully moved out of local optima based on the balance in the search procedure. Another enhanced SMA (ESMA) was reported in^64^ and is like the preceding variant with respect to the use of chaotic theory but differs from its use of the opposition learning method. ESMA was applied to solve the estimation of water needs in a region using data on historical water consumption and local economic structure. In^65^, opposition learning and the method of weight coefficient adaptation are also used to design a new, improved variant (ISMA). On the other hand, Izci^66^ addressed the local search concentration of SMA using Nelder-Mead (NM) simplex search to obtain SMA-NM. The added method achieved the desired balance between exploration and exploitation. Recently, NafiÖrnek^67^ proposed a new variant which improves the oscillation process of SMA using different transformations of the sine cosine algorithm. ArcTanh function has been replaced using the sigmoid function, and the sine cosine algorithm now computes a new position for SMA. In another study, Abdel Basset^68^ created a new variant through a hybrid of SMA with a Whale Optimization Algorithm. The version of SMA reported in^69^ adapted the algorithm to the chaotic theory, which uses Chebyshev mapping. In^70^, SMA’s weakness in balancing exploration and exploitation was addressed using an Adaptive Opposition Slime Mould Algorithm (AOSMA), using adaptive learning, so that one random search agent is replaced with the best updating position. In^71^, an improved SMA (ISMA) does not escape the best solution to solve the problem of Optimal Reactive Power Dispatch (ORPD) to guarantee the reliability and economy of a power system.

Considering all these biology-based optimization algorithms and their associated variants, we deduced that performance of metaheuristic algorithms could be improved by looking not only at their strength derivable from nature but addressing weaknesses inherited from nature. As a result, this study seeks to investigate the inherited weaknesses of EOSA as they relate to its source of inspiration, and then design and enhance the version.

Meanwhile, hybrid methods of metaheuristic algorithms have also been proposed to obtain new variants demonstrating high-performing optimization results. For example, Aquila Optimizer (AO) and Arithmetic Optimization Algorithm (AOA) have been proposed to solve problems of high-dimensional and low-dimensional nature^72^. Similarly, Hunger Games Search (HGS) and Arithmetic Optimization Algorithm (AOA) have been hybridized to solve unconstrained and constrained problems of both high- and low-dimensional nature^73^. Drawing inspiration from the fertility nature of farmland, a new optimization algorithm has been proposed named Farmland Fertility Algorithm (FFA). The new algorithms uniquely optimize optimization problems so that solutions’ internal and external memory are considered. An exhaustive evaluation of the algorithm showed that it decreases significantly in terms of dimensions^74^. New algorithm variants have also been proposed with discrete metropolis acceptance criterion^75^, and a modified version for solving constrained engineering problems^76^. In another related study, authors have proposed a metaheuristic algorithm based on the behavior of African vultures. The new algorithm, named African Vultures Optimization Algorithm (AVOA), leverages the foraging and navigation attributes of this creature^77^. Also, the behavior of gorilla troops and their social intelligence have motivated the design of an optimization algorithm, namely Artificial Gorilla Troops Optimizer (GTO). The novel GTO is a model based on the collective lifestyle of gorillas^78^. Similarly, a recent metaheuristic algorithm named Prairie Dog Optimization (PDO) has been proposed to solve classical benchmark functions and real-life optimization problems. The algorithms were inspired by the natural behavioral pattern of the prairie dogs with respect to their foraging and burrowing lifestyle^79^.

### Feature extraction and optimization in deep learning

The solution to the challenge of feature extraction has been modeled using deep learning (DL) models. However, knowing which subset of features is suggestive of the existence of abnormality, leading to solving the classification problem, remains a challenge. In this section, we present a review of studies that have attempted to address this problem. The study in^23^ applied metaheuristic algorithms to improve the selection of preferred detected features leading to the classification of the presence of White Blood Cell (WBC) Leukaemia in samples. Using CNNs to extract features, the Salp Swarm Algorithm (SESSA) was applied to filter out suggestive features, while highly correlated and noisy features were eliminated. The study revealed that only 1000 of 25,000 features proved relevant to the classification task. Also, Fatani et al.^24^ used the traditional method of CNN extracting features and then applied Aquila Optimizer (AQU) for further feature selection. The performance of the new approach was investigated using CIC2017, NSL-KDD, BoT-IoT, and KDD99 datasets. In^25^, Grasshopper Optimization Algorithm and the Crow Search Algorithm were hybridized to address the challenge of feature selection leading to classification using MLP. Results showed that when combined with MLP, the hybrid method returned the values of 97.1%, 98%, and 95.4% for accuracy, sensitivity and specificity, respectively, using a mammographic dataset. In another study^29^, Advanced Squirrel Search Optimization Algorithm (ASSOA) was used to scale down the number of detected features from the CNN (ResNet-50, specifically) phase, so that the classification task could follow with reduced effort. The hybrid method was further supported by using image augmentation to improve the performance of the whole process. In addition, the study investigated the performance of Multilayer Perceptron (MLP) Neural Networks when ASSOA is applied to optimize weights. Results showed that both ASSOA and MLP returned a classification mean accuracy of 99.26%.

While studies which have advanced research in this way are limited, several have limited their works only to using features extracted by CNN. For example, Jogin et al.^80^ extracted features to support classification using CNN, while^81^ optimized the parameters of the CNN using a metaheuristic algorithm, namely Manta-Ray Foraging-Based Golden Ratio Optimizer (MRFGRO), to improve performance so that better features are extracted solely by the CNN architecture. The work in^82^ experimented with using medical images of the chest, lung, brain, and liver to investigate how the fusion of extracted features would improve the accuracy of classifying abnormalities in the domain. So, the method advances the norm using the fusion technique. Authors in^83^ suggested the use of the CNN-based auto features extraction (CNN-AFE) method to enhance classification functions. Features extracted using CNN were further classified using a shallow CNN architecture. Other studies, like^84^, have proposed using staked CNN two-stream deep architecture as an example and dimensionality reduction in achieving refined feature extraction leading to classification. The classification was achieved using Support Vector Machine (SVM) concatenated features from previous methods. Similarly, Lu et al.^85^ uses feature-fusion improved features resulting from feature extraction using a deep convolutional neural network (DCNN). The concept worked by fusing features resulting from the two nets.

Dimensionality reduction methods such as PCA are often applied to filter relevant features. The SVM result showed that the method yielded 89.24% and 97.19% for recall and precision, respectively. In our recent study^86^, we demonstrated the use of the wavelet function to optimize the selection process of suggestive features leading to classification accuracy. A hybrid algorithm involving seam carving and wavelet decomposition was experimented with for image preprocessing to find discriminative features. These filtered features are passed as input to a CNN-wavelet structure for further feature extraction and then classification using DDSM + CBIS and MIAS datasets. Using a novel technique for improved performance for high dimension datasets, a study proposed complex cisoidal (cisoid) analysis based on feature selection (CAFS). The approach leverages the ability to relate members of feature space and pre-established methods to complex sinusoids using a mathematical model. The resulting method yielded better classification accuracy^87^.

The use of metaheuristic algorithms to aid feature selection on non-image level inputs has also been reported. However, the focus of this study is to derive a novel approach to support the use of metaheuristic algorithms in further refining extracted features from CNN operations. As a result, in this study, we first proposed an improved version EOSA method and then applied the new variant to filter down extracted features resulting from convolutional operations of CNN. To enhance performance, the aim was to improve classification accuracy and avoid bottle-necked classification functions. This method is elaborated and implemented for performance evaluation with state-of-the-art methods in the following sections.

## Methodology

The Immunity-Based Ebola Optimization Search Algorithm (IEOSA) is presented in this section. We detail our design based on the optimization process, mathematical model, the procedure for the optimization process, the flowchart, and then the algorithm. Thereafter, the applicability of the IEOSA to solving the challenging problem of minimization of the density of extracted features from the convolutional-pooling blocks is demonstrated through an adapted approach following some mathematical models. A modified CNN architecture from our recent study^73^ is presented to demonstrate that applicability but is now coupled with the optimization process. The design method described in this section aims to improve the convergence rate, and enhance the search strategy of the optimization algorithm so that it can escape local optima and avoid missing the best solution in local space. The population in the search space follows the chaotic theory and mimics the natural immunity residual in the human body in fighting the Ebola virus.

### Inspiration-derived optimization process

**Figure 1:**
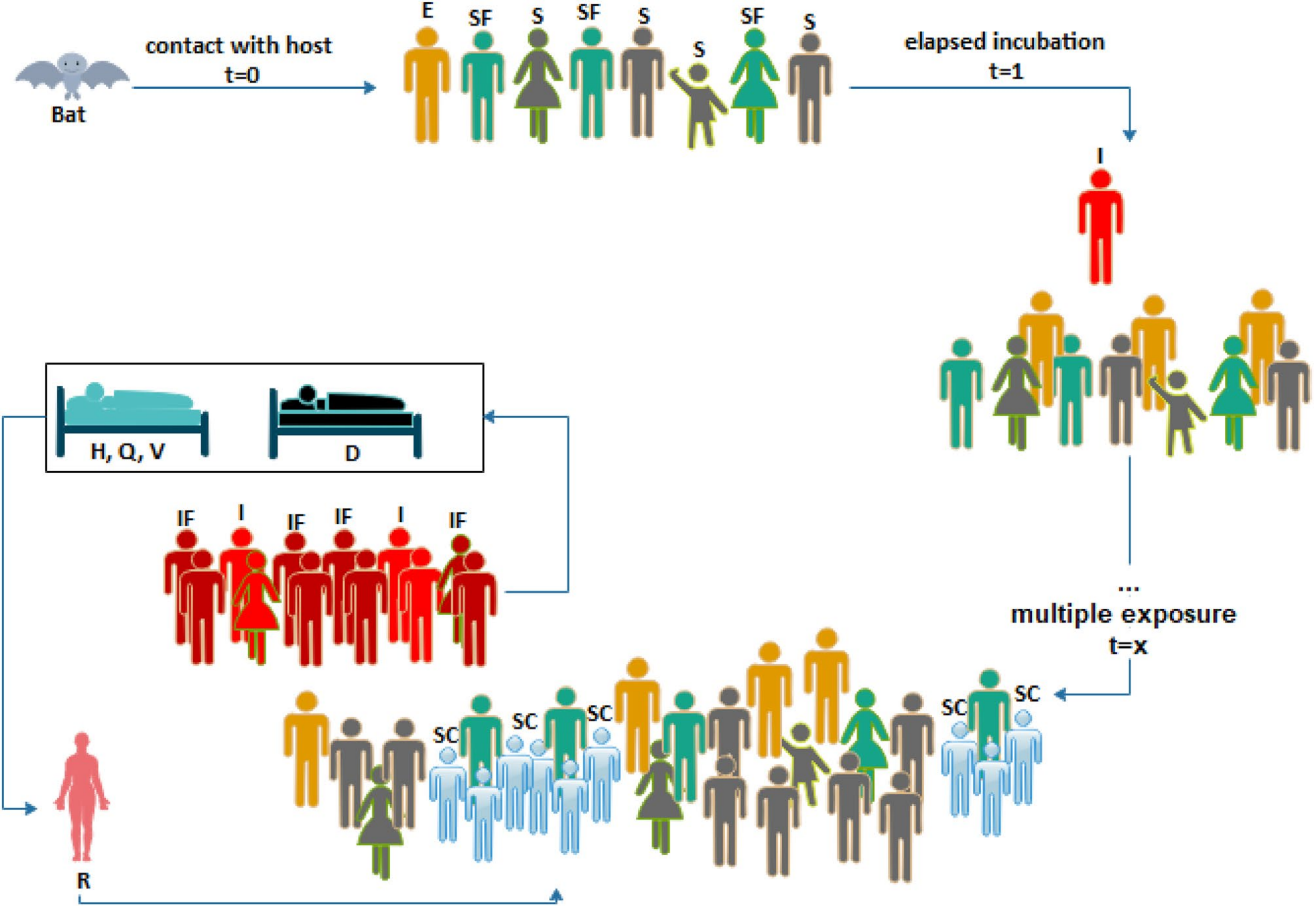
Optimization process based on the Immunity-Based Ebola Optimization Search Algorithm. The model advances EOSA with three (3) subgroups, namely Infected with Immunity Factor (IF), Susceptible with Immunity Factor (SF), and Susceptible Covered by Immunity (SC).

The demonstration of the optimization process is illustrated in Fig. 1, which shows all the subgroups in the population from time $t=0$ to an arbitrary time $t=x$. The model leverages the SIR model presented in our recent study^20^ to unwrap each subgroup in the population into their role in achieving the aim of this study. In addition to well-defined Susceptible (S), Infected (I), Recovered (R), Hospitalized (H), Quarantined (Q), Vaccinated (V), and Dead (D) subgroups, others like Infected with Immunity Factor (IF), Susceptible with Immunity Factor (SF), and Susceptible Covered by Immunity (SC) are described.

Studies have proven that the virus enters the human population through exposure to a host agent or infested environment so that such an exposed individual becomes the index case once an estimated incubation period elapses^88^. The index case propagates the virus among members of the susceptible group so that more infected cases are recruited. Considering the differences in the composition of solutions represented in individuals, based on the membership of these subgroups and the propagative nature of the virus among subgroups, the optimization process becomes interesting and useful. The composition of each potential solution allows for representing new subgroups, namely IF, SF, and SC, with a much more diversified composition, thus presenting the search processes with more viable and promising solutions in the search space. Table 1 lists some parameters supporting the rates at which individuals are propagated across subgroups.

**Table 1:** Notations and description for variables and parameters for SEIR-HDVQ.

Symbol	Description	Symbol	Description
π	Recruitment rate	γ	Recovery rate
ŋ	Decay rate of EVD	τ	Natural death rate
α	Hospitalization rate	δ	Burial rate
Γ	Disease-induced death rate	ϑ	Vaccination rate
β_1_	Contact rate of infected	ϖ	Treatment rate
β_2_	Contact rate of host	μ	Response rate
β_3_	Contact rate with the dead	ξ	Quarantine rate
β_4_	Contact rate with the recovered	γ	Recovery rate

### Mathematical model of optimization process

The mathematical model described in this section models the population initialization, allocation of sub-population groups, mutation of infected individuals, exploration and exploitation stages,

#### Initialization of population

The initialization of the individual in the population of IEOSA follows the logistic chaotic method. This is motivated by the nature of the optimization problem addressed in this study, as supported by^89^ and^90^, and the method’s tendency to improve performance based on diversity of population and convergence speed. In the beginning, an initial population is generated utilizing random number distribution so that the zero (0) individual is generated, as shown in Eq. (1). The ***U*** and ***L*** denote the upper and lower bounds, respectively. Representation of all individuals follows Eq. (2), where *i* = 1,2,3…., N, *d* = 1, 2, D, with N and D denoting the population size and dimension, respectively. Subsequent individuals in the population are derived using Eq. (3).

1 $${ind}_{i}=L+rand\left(\mathrm{0,1}\right)*\left( U - L\right)$$

2 $$individual=\left[\begin{array}{ccc}{ind}_{\mathrm{1,1}}& \cdots & {ind}_{1,d}\\ \vdots & \ddots & \vdots \\ {ind}_{1,n}& \cdots & {ind}_{n,d}\end{array}\right]$$

3 $${ind}_{i+1}=\mathfrak{g}* {ind}_{i} *\left(1- {ind}_{i}\right)$$

where $\mathfrak{g}$ was experimentally investigated on the values of 3 and 4, the selection of the current best is computed on the set of infected individuals in time t, as seen in Eq. (4):

4 $${ind}_{best}=\left\{\begin{array}{c}{ ind}_{best}, fitness({ ind}_{cbest})<fitness\left({ ind}_{best}\right)\\ { ind}_{cbest}, fitness\left({ ind}_{cbest}\right)\ge fitness\left({ ind}_{best}\right)\end{array}\right.$$

#### Selection of SF, IF, and C individuals

Susceptible with Immunity (SF), Infected with Immunity (IF), and Susceptible Covered by Immunity (SC) are selected from their respective populations and generated using Eqs. (5), (6) and (7).

5 $$SF= \frac{1}{3}* size(S)$$

6 $$SC= \frac{1}{16}* size(S- SF)$$

7 $$IF= \frac{1}{4}* size(I)$$

#### Immunity factor (IM)

The immune individuals can be found both among the infected and the susceptible. When found susceptible, they can remain asymptomatic with the disease and will show some symptoms when found as infected but with little spreading rate, due to lowered strength of the disease in them. $IM$ represents the immunity vector computed using Eqs. (8) and (9) for SF and IF, respectively:

8 $${IM}_{i}={B}_{1}*{(ind}_{{S}_{best}}-{ ind}_{i}) + {ind}_{i}$$

9 $${I^{\prime}M}_{i}={{B}_{2}*(ind}_{Ibest}- {ind}_{i}) + {ind}_{i}$$

where immunity benefit vectors ${B}_{1}$ and ${B}_{2}$ are computed using $\frac{{ind}_{Sbest} - {ind}_{Sworst}}{2}$ and $\frac{{ind}_{Ibest} - {ind}_{Iworst}}{2}$ respectively.

#### Mutation of infected (I)

The impact of infection on an individual solution reduces its quality, hence the representation of the mutation operation in Eq. (10):

10 $${ind}_{i}= {e}^{rand(-1, 1)}cos2\pi *rand(-1, 1) + {ind}_{i}$$

#### Position update

TO update the positions of each exposed individual, Eq. (11) applies:

11 $${mI}_{i}^{t+1}={mI}_{i}^{t}+rand\left(-1\left|0\right|1 \right)*\uprho$$

where ρ represents the scale factor of displacement of an individual, ${mI}_{i}^{t+1}$ and ${mI}_{i}^{t}$ are the updated and original position at time t and t + 1, respectively. The $rand\left(-1\left|0\right|1\right)$ randomly yields a value which can be -1 or 0 or 1, with each denoting movement leading to covered, intensification, and exposed displacements, respectively.

#### Update of infected (I) and susceptible (S)

For an arbitrary time $t$, the number of individuals added to $I$ and $S$ is computed based on the natural representation of the disease. These updates are represented by Eqs. (12) and (14), while their impact on S is modeled in Eqs. (13) and (15), respectively:

12 $${newS}_{t}=\frac{\partial S(t)}{\partial t}=\pi-\left({\upbeta }_{1}\mathrm{I}+ {\upbeta }_{3}\mathrm{D}+ {\upbeta }_{4}\mathrm{R}+ {\upbeta }_{2}\left(\mathrm{PE}\right) {\upeta}\right)S-(\mathrm{\tau S}+\mathrm{\Gamma I})$$

13 $$S=S+{newS}_{t}$$

14 $${newI}_{t}=\frac{\partial I\left(t\right)}{\partial t}=\left({\upbeta }_{1}\mathrm{I}+ {\upbeta }_{3}\mathrm{D}+ {\upbeta }_{4}\mathrm{R}+ {\upbeta }_{2}\left(\mathrm{PE}\right)\uplambda \right)S-\left(\Gamma +\upgamma \right)I -\left(\uptau \right)\mathrm{S}$$

15 $$I=I+{newI}_{t}$$

#### Exploration phase

Two factors influence increased contagion and infection with the disease. These are the displacement rate $lrate$ of an infected (I) and the rate of occurrence of superspreading events $supersrate$ among susceptible (S). To apply these factors to the population, a random number of infected individuals (I) are exposed to a random number of susceptibles (S), guided by $lrate$ and $supersrate$. The parameters $supersrate$ and $lrate$ increase linearly with time $t$, from -1 to 1 and 0.5 to 2, respectively, as seen in Eq. (16).

16 $${newI}_{t}= \lceil I * lrate*rand \rceil+ \lceil S* supersrate*rand \rceil$$

#### Exploitation phase

Like exploration, two major factors have been reported to help mitigate the spread of the virus. These are social distancing $socialdrate$ among the susceptible (S) and quarantining of infected (I) $srate$. The parameters $socialdrate$ and $srate$ decrease and increase with time $t$, from 2 to 0 and 0 to 1 linearly, respectively, as seen in Eq. (17).

17 $${newI}_{t}=\lceil I* srate*rand\rceil+ \lceil S* socialdrate *rand\rceil$$

The use of $socialdrate$ and $supersrate$ variables is supported by data indicating direct physical contact and exposure to infected body fluids, as primary modes of Ebola virus transmission^91^. Note that $rand$ generates random number in the range [0, 1]. Infection resulting from contagion at the exploration or exploitation stages impacts the current number of susceptible (S) as shown in Eq. (18):

18 $$S=S - {newI}_{t}$$

#### Update of recovered (R)

Recovered individuals from infected (I) cases are obtained using Eq. (19) in time $t$. The mutation of solutions in R is evaluated by Eq. (20) for individuals with immunity (IF) and Eq. (21) for non-immune individuals. The notation $I^{\prime}M$ represents residual immunity after recovery from the disease and applies to each individual ${ind}_{i}$ in R.

19 $$\frac{\partial R(t)}{\partial t}=\upgamma I-\Gamma R$$

20 $$S=S+ R$$

21 $$S=S+(R*I^{\prime}M)$$

#### Update of dead (D)

Naturally, new births are recorded as the population is affected by natural death and death through the virus. The death rate due to the virus is computed using Eq. (22), and updates on S due to D and the new births are represented using Eq. (23).

22 $$\frac{\partial D\left(t\right)}{\partial t}=\left(\mathrm{\tau S }+\mathrm{\Gamma I}\right)-\updelta D$$

23 $$S=S - D+(rand*S)$$

#### Compartment H, V, and Q update

The application of differential calculus, in our case, intends to obtain the rates of change of quantities H, V D, and Q with respect to time $t$. Hence, the Eqs. (24)–(26) are as follows:

24 $$\frac{\partial H(t)}{\partial t}=\mathrm{\alpha I}-(\upgamma +\mathrm{\varpi })\mathrm{H}$$

25 $$\frac{\partial V(t)}{\partial t}=\upgamma I -(\upmu +\mathrm{\vartheta })V$$

26 $$\frac{\partial Q(t)}{\partial t}=\left(\mathrm{\pi I}-(\mathrm{\gamma R}+\mathrm{\Gamma D}\right))-\upxi Q$$

We assume that Eqs. (12–24) are *scalar functions*, meaning that they have one number as a value, which can be represented as a float.

### Design of IEOSA

The design of the Immunity-Based EOSA (IEOSA) metaheuristics algorithm follows from the listing of the procedure for the pseudocode to the design of the flowchart and then the algorithm design. We combine the optimization process and the mathematical models to achieve the design described in this section. The formalization of the IEOSA algorithm is according to the procedure outlined in the following:

1. Initialize all parameters and assign population (where necessary) to all compartments, namely Susceptible (S), Infected (I), Recovered (R), Dead (D), Vaccinated (V), Hospitalized (H), and Quarantined (Q), Susceptible with Immunity (SF), Infected with Immunity (IF) and Susceptible Covered by Immunity (SC).
2. Compute fitness of all individuals in S.
3. Set the best individual in S as the index case.
4. Make the index case the global best and current best.
5. While the condition for termination is not satisfied and there exists at least an infected individual, then
  - Quarantine a fraction of I
  - For each remaining infected individual:
    i. Compute the new position of the current individual based on randomized displacement.
    ii. Expose only a fraction of S to the current individual.
    iii. Generate newly infected individuals and mutate their solution.
    iv. Add the newly generated cases to the I.
  - Compute the number of individuals to be added to H, D, R, D, V, and Q, using corresponding equations based on the I.
  - Update S with new infections.
  - Select the current best from I and compare it with the global best.
  - Go back to step 5.
6. Return global best solution.

**Figure 2:**
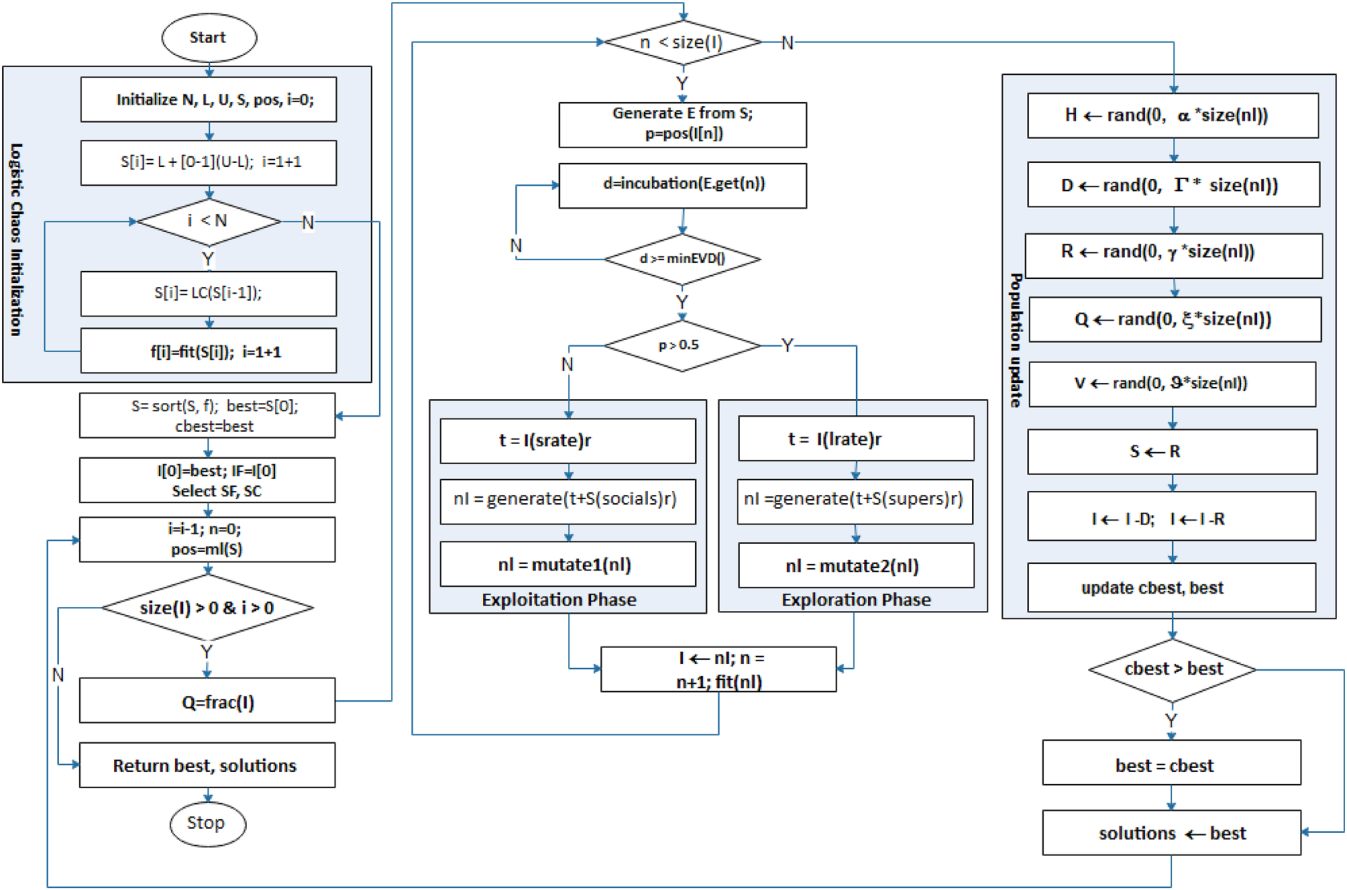
Flowchart demonstrating the optimization process of the improved IEOSA from the initialization and search process to the return of the best solution obtained during the training.

In Fig. 2, the flow chart of the improved IEOSA metaheuristic algorithm is illustrated.
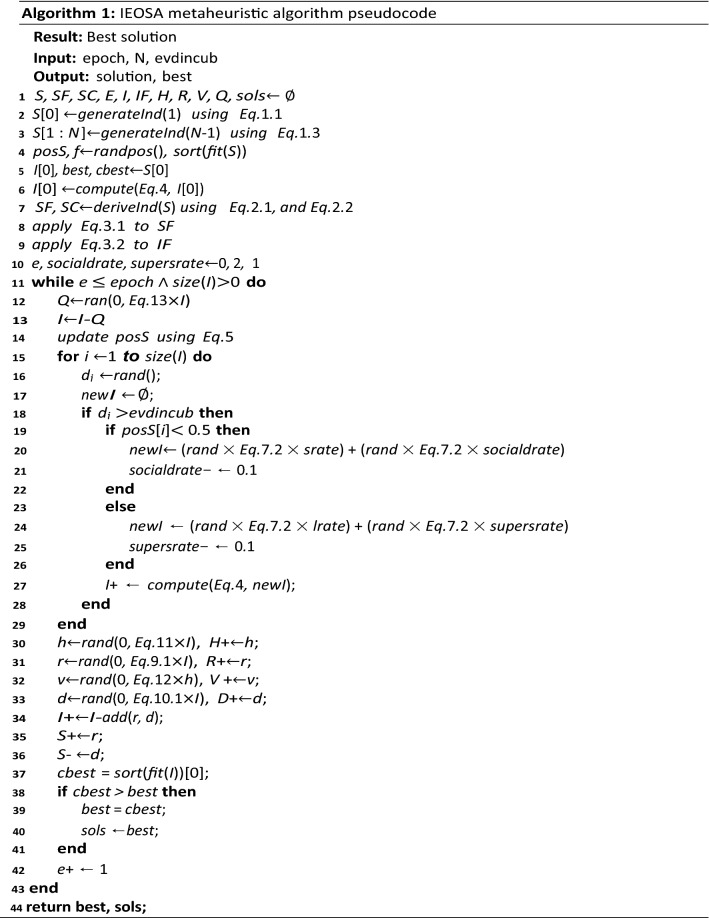

In Algorithm 1, the translation of the procedures and flowchart into algorithmic solutions is described. Listings on lines 1–7 describe the initialization of relevant parameters discussed in previous subsections. The mutation of the composition of solutions represented in SF and IF are described in lines 7–8. Initialization of control parameters such as the $e$, $socialdrate$ *and* $supersrate$ is done on line 10. Lines 11–43 list the lines describing the training of the IEOSA where solutions are mutated, and the search for the best solution is carried out within the search space. The exploration and exploitation stages of the algorithm are captured on lines 19–22 and 23–26, respectively. Lines 30–36 demonstrate all subgroups’ updates using their corresponding control rates listed in Table 1. The current best solution is computed based on the fitness function, and the update for the global best is done on lines 37–41. The iteration control variable is incremented on line 42, so that satisfiability of termination condition is evaluated on 11, failure of which leads the algorithm flow to line 44, where the best solution and all relevant solutions are returned for use to address the optimization problem.

### IEOSA feature extraction optimization

The adaption of the IEOSA metaheuristic algorithm to address the optimization problem related to the minimization of the number of features extracted using CNN architectures is presented in this section. The minimization is guided by the need to select only suggestive features with discriminant capabilities supporting the process of classification of abnormalities in digital mammography.

#### Architecture of the CNN and feature extraction

**Figure 3:**
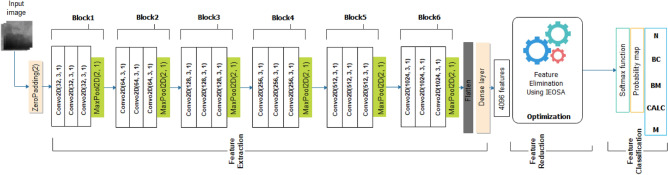
An illustration of the design of the CNN-IEOSA model consisting of the feature extraction, feature reduction and feature classification compartments. The CNN has six (6) convolutional-pooling blocks that output extracted features using a dense layer.

The architecture of the CNN consists of six (6) blocks of convolutional-pooling layers that provide for feature extraction. Each block comprises three (3) convolutional operations, followed by a max-pooling operation. The number of filters used for each of the three convolution layers in all blocks is a 3 × 3 size. The filter count for all layers in each block of the six (6) blocks follows the 32, 64, 128, 256, 512, and 1024 series. Input to the first layer of the first block is prepended with a zero-padded layer which uses a 1 × 1 size so that inputs to the architecture, which assumes a 299 × 299 pixel size, are padded with zeros for better convolutional operation. All convolutional layers use the RELU activation function and kernel regularizer of L1 at the rate of 0.0002. The max-pooling layers in each block are built with a 2 × 2 size and 2 × 2 strides, to allow for pooling out overwhelming features. The 6-convolutional-pooling-block is followed by a flatten layer, which vectorizes the extracted features before passing it to a dropout layer, which applies a 0.5 rate and a dense layer using a 4096 size for preparing the extracted features for input to the IEOSA optimization process. Meanwhile, the batch size used for training is 32, using the softmax function for the classification. The evaluation of loss values is achieved using the categorical cross-entropy function. The training uses 100 epochs with a learning rate of 1e-05 and the Adam optimizer with the configuration of: beta_1 = 0.9, beta_2 = 0.999, epsilon = 1e-8. Each image passed through the CNN architecture returns a feature size of 4096 (per image) and is scaled down by the IEOSA optimizer so that only discriminant features with non-bottleneck features are returned for classification with the softmax function. Figure 3 illustrates the CNN architecture described in this section, showing that five classes are used for the classification problem. Each image feature returns a probability map indicating what class the image belongs to.

#### Feature optimization with CNN

The formulation of the feature elimination process using the IEOSA metaheuristic algorithm is described in this section. Consider that the optimization process aims to obtain a subset of features from those representing an image, so that the minimum number of features suitable to support the classification task optimally is reported. The sub-population represented by the susceptible (S) represents all features obtained for an arbitrary image $i$ denoted with Eq. (27).

27 $$image[i]=\left[\begin{array}{ccc}{feature}_{\mathrm{1,1}}& \cdots & {feature}_{1,d}\\ \vdots & \ddots & \vdots \\ {feature}_{1,n}& \cdots & {feature}_{n,d}\end{array}\right]$$

A feature is selected as the index case so that it is added to the infected (I) subgroup. The features are optimised over a few iterations by dynamically moving them across all sub-populations until the best subset of features representing the most suggestive for the classification task are grouped in the I population, which are defined using Eq. (28).

28 $${feature}_{i}= {e}^{rand(-1, 1)}cos2\pi *rand(-1, 1) + {feature}_{i}$$

Evaluation of the fitness of the subsets of features represented by each infected case in I is computed using Eq. (30), which represents the softmax classifier function, and Eq. (29) which is the classification accuracy.

29 $${\sigma \left(\overrightarrow{x}\right)}_{i}=\frac{{{e}}^{{\overrightarrow{x}}_{{i}}}}{{\sum }_{{j}=1}^{n}{{e}}^{{\overrightarrow{x}}_{{j}}}}$$

30 $$f\left(t\right)=accuacy=\frac{TP+TN}{\left(TP+TN+FP+FN\right)}$$

where the output $\overrightarrow{x}$ is the vector representation of extracted features which is passed to the softmax function, ${\mathcal{e}}^{{x}_{\mathcal{i}}}$ is the standard exponential function applied to each element of the input vector, and $n$ is the number of classes in the multi-class classifier.

## Experimentation setup

A detailed description of the setup of parameters, computation environment, and measures for computation of evaluation performance is presented in this section.

### Dataset and computation environment

Python 3.7.3 and all supporting libraries such as Tensorflow, Keras, and other dependent libraries were used to set up the environment for the experimentation. A computer workstation with each configuration was used: Intel (R) Core i5-4200, CPU 1.70 GHz, 2.40 GHz; RAM of 8 GB; 64-bit Windows 10 OS. This same system with the indicated configuration was used for testing the trained model.

Experimentation with the dataset combining those from the Mammographic Image Analysis Society (MIAS)^92^ and the Curated Breast Imaging Subset (CBIS) of the Digital Database for Screening Mammography (DDSM + CBIS)^93^ was applied to the method described in this study. The two datasets have samples belonging to one of the following classes: normal (N), benign with calcification (BC), benign with mass (BM), calcification (CALC), and mass (M). Image samples from the datasets were preprocessed using the CLAHE method for improved CNN model input. A total of 3075 samples were sourced from the MIAS dataset, while a total of 55,904 samples were sourced from the DDSM + CBIS dataset. The combined dataset yielded a total of 58,979 samples, which were further allocated for training, evaluation, and testing at the rate of 75%, 15% and 10%, respectively. This distribution of samples was carried out to achieve a balanced image class label. Class-based enumeration for samples resulted in 51, 453, 1868, 1767, and 1961 for the N, BC, BM, CALC, and M labels, respectively.

**Figure 4:**
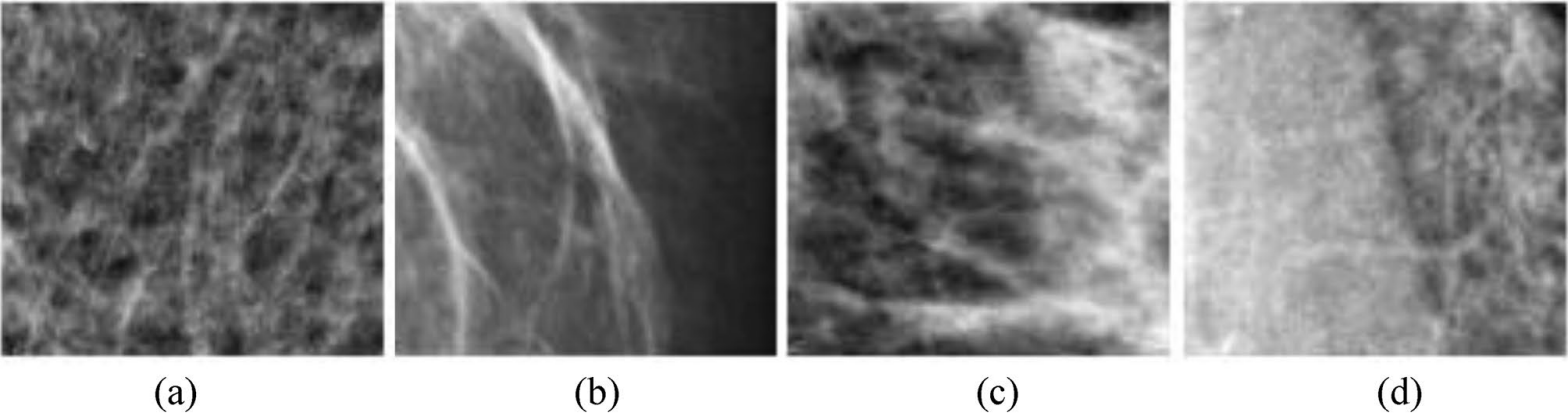
Samples of images to be extracted from combined datasets sourced from DDSM + CBIS and MIAS databases. Image labels: (**a**) mass abnormality (M); (**b**) calcification abnormality (CALC); (**c**) benign calcification (BC); and (**d**) benign with mass.

**Figure 5:**
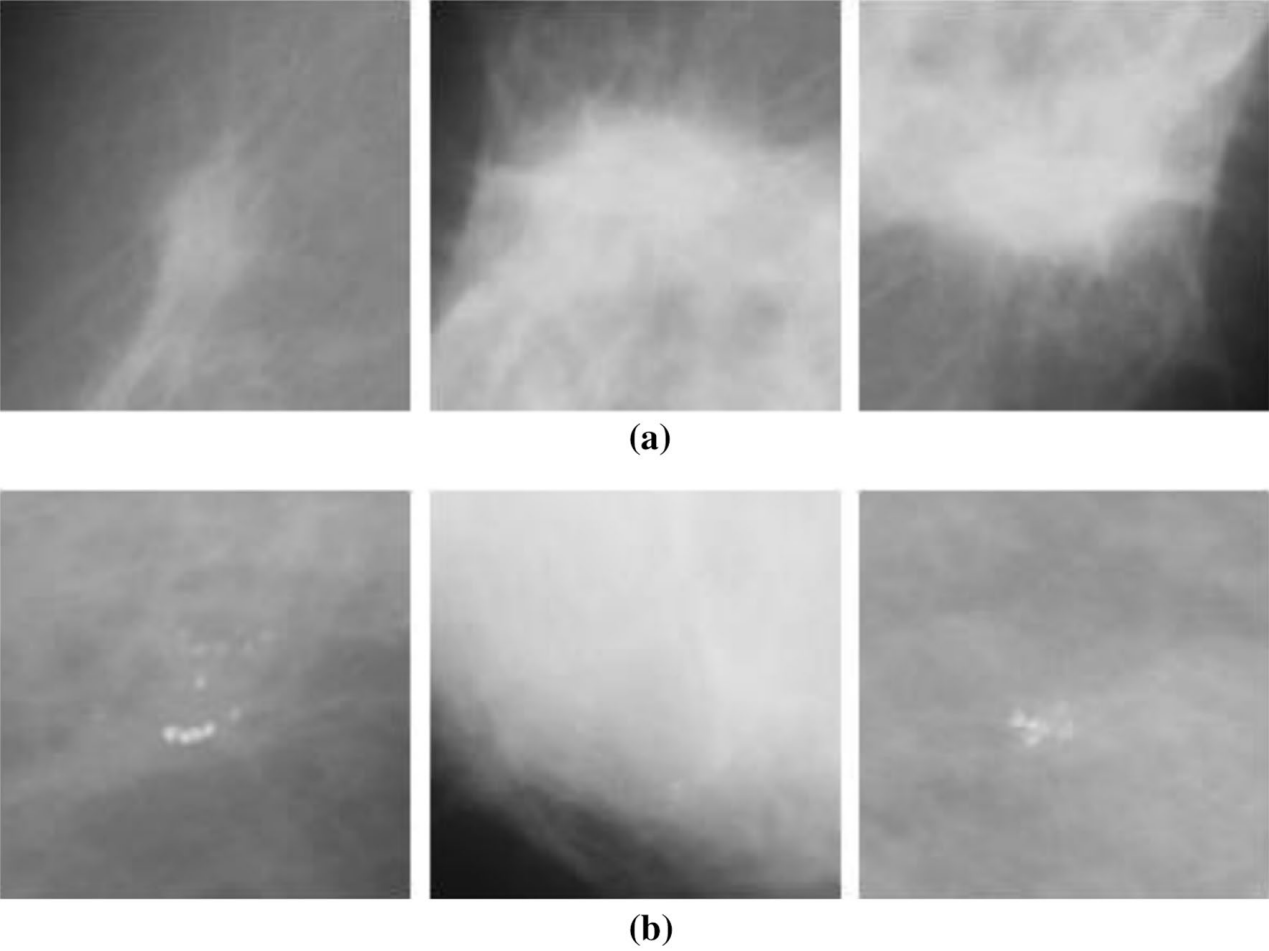
(**a**) Samples with different orientations of calcification abnormalities from the combined datasets from DDSM + CBIS and MIAS databases. (**b**) Samples with different orientations of mass abnormalities from the combined datasets from DDSM + CBIS and MIAS databases.

Figure 4 presents a graphical illustration of the distribution of image samples from the DDSM + CBIS and MIAS datasets. The figure shows samples with mass abnormality (M), calcification abnormality (CALC), benign calcification (BC), and benign with mass, in their respective orders. A further presentation of more samples showing different variations and orientations of abnormalities containing mass and calcification is seen in Fig. 5 (a-b).

### Configuration parameters and benchmark functions

The experimentation for the IEOSA method was evaluated using a population size of one hundred (100), and the training epoch of five hundred (500) was used. Each 500-epoch training was executed twenty (20) times to ensure a normal performance of the experiment. Tables 2 and 3 present an outline of all the benchmark functions used for experimentation and evaluation of the improved IEOSA method.

**Table 2:** Classical benchmark functions used to evaluate the IEOSA method, where Dimension (D), Multimodal (M), Non-separable (N), Unimodal (U), and Separable (S) describe entry in the “Type” column.

ID	Function name	Model of the function	Type
F1	Sum-POWER	$\mathrm{f}(\mathrm{x})=\sum_{\mathrm{i}=1}^{\mathrm{n}}{\mathrm{ix}}_{\mathrm{i}}^{2}$	US
F2	Brown	$\mathrm{f}\left(\mathrm{x}\right)=\sum_{\mathrm{i}=1}^{\mathrm{n}-1}{{({\mathrm{x}}_{\mathrm{i}}^{2})}^{\left({\mathrm{x}}_{\mathrm{i}+1}^{2}+1\right)}+({\mathrm{x}}_{\mathrm{i}+1}^{2})}^{({\mathrm{x}}_{\mathrm{i}}^{2}+1)}$	UN
F3	Dixon and price	${\mathrm{f}}_{18}\left(\mathrm{x}\right)={10}^{6}{\mathrm{x}}_{1}^{2}\sum_{\mathrm{i}=2}^{\mathrm{D}}{\mathrm{x}}_{\mathrm{i}}^{2}$	UN
F4	Generalized penalized function	$\mathrm{f}\left(\mathrm{x}\right)=0.1\mathrm{ X }\left\{{\mathrm{sin}}^{2}\left(3\uppi {\mathrm{x}}_{1}\right)+ {\sum }_{\mathrm{i}=1}^{\mathrm{n}-1}{\left({\mathrm{x}}_{\mathrm{i}}-1\right)}^{2} \left[1+{\mathrm{sin}}^{2}\left(3\uppi {\mathrm{x}}_{\mathrm{i}+1}\right)\right]+{\left({\mathrm{x}}_{\mathrm{n}}-1\right)}^{2}[1+{\mathrm{sin}}^{2}(2\uppi {\mathrm{x}}_{\mathrm{n}})]\right\}+{\sum }_{\mathrm{i}=1}^{\mathrm{n}}\mathrm{u}\left({\mathrm{x}}_{\mathrm{i}},\mathrm{ a},\mathrm{ k},\mathrm{ m}\right)$ Where $$\mathrm{u}\left({\mathrm{x}}_{\mathrm{i}},\mathrm{ a},\mathrm{ k},\mathrm{ m}\right)= \left\{\begin{array}{c}k{({\mathrm{x}}_{\mathrm{i}}-\mathrm{a})}^{\mathrm{m}} if {\mathrm{x}}_{\mathrm{i }}>a\\ 0 if-a \le {\mathrm{x}}_{\mathrm{i}} \le a\\ k{(-{\mathrm{x}}_{\mathrm{i}}-\mathrm{a})}^{\mathrm{m}} if {\mathrm{x}}_{\mathrm{i}< -\mathrm{a}}\end{array}\right.$$ a = 5, k = 100, m = 4	M
F5	Inverted cosine mixture	${\mathrm{f}}_{14}\left(\mathrm{x}\right)= 0.1\mathrm{n }- \left(0.1 \sum_{\mathrm{i}=1}^{\mathrm{n}}\mathrm{cos}(5\uppi {\mathrm{x}}_{\mathrm{i}})- \sum_{\mathrm{i}=1}^{\mathrm{n}}{\mathrm{x}}_{\mathrm{i}}^{2}\right)$	MS
F6	Noise	${\mathrm{f}}_{7}\left(\mathrm{x}\right)=\sum_{\mathrm{i}=1}^{\mathrm{n}}{\mathrm{x}}_{\mathrm{i}}^{4}+\mathrm{random}[0, 1)$	N
F7	Pathological function	$\mathrm{f}\left(\mathrm{x}\right)=\sum_{\mathrm{i}-1}^{5}\mathrm{icos}\left(\left(\mathrm{i}-1\right){\mathrm{x}}_{1}+\mathrm{i}\right)\sum_{\mathrm{j}=1}^{5}\mathrm{jcos}\left(\left(\mathrm{j}+1\right){\mathrm{x}}_{1}+\mathrm{j}\right)$	MN
F8	Powel	$\mathrm{f}\left(\mathrm{x}\right)={\left({\mathrm{x}}_{1}+10{\mathrm{x}}_{2}\right)}^{2}+5{({\mathrm{x}}_{3}+{\mathrm{x}}_{4})}^{2}+{({\mathrm{x}}_{2}- {2\mathrm{x}}_{3})}^{4}+10{({\mathrm{x}}_{1}-{\mathrm{x}}_{4})}^{4}$	UN
F9	Rastrigin	${\mathrm{f}}_{9}\left(\mathrm{x}\right)=\sum_{\mathrm{i}=1}^{\mathrm{n}}[{\mathrm{x}}_{\mathrm{i}}^{2}-10\mathrm{cos}\left(2\uppi {\mathrm{x}}_{\mathrm{i}}\right)+10]$	MN
F10	Rotated hyperellipsoid	${\mathrm{f}}_{3}\left(\mathrm{x}\right)=\sum_{\mathrm{i}=1}^{\mathrm{n}}\left(\sum_{\mathrm{j}=1}^{\mathrm{i}}{\mathrm{x}}_{\mathrm{j}}\right)$	U
F11	Schwefel 2.26	$\mathrm{f}(\mathrm{x})=\sum_{\mathrm{i}=1}^{\mathrm{n}}\left[-{\mathrm{x}}_{\mathrm{i}}\mathrm{ sin}(\sqrt{{|\mathrm{x}}_{\mathrm{i}}|})\right]$	MS
F12	Sphere	${\mathrm{f}}_{1}\left(\mathrm{x}\right)=\sum_{\mathrm{i}=1}^{\mathrm{n}}{\mathrm{x}}_{\mathrm{i}}^{2}$	US
F13	Shift-rotated of sum of different power	${\mathrm{f}}_{21}\left(\mathrm{x}\right)=\mathrm{SR}(\sum_{\mathrm{i}=1}^{\mathrm{d}}{{|\mathrm{x}}_{\mathrm{i}}|}^{\mathrm{i}+1} )$	N
F14	Shifted and rotated Zakharov function	$\mathrm{f}(\mathrm{x})=\mathrm{SR}(\frac{1}{\mathrm{n}}\sum_{\mathrm{i}=1}^{\mathrm{n}}1-\mathrm{cos}\left(10{\mathrm{x}}_{\mathrm{i}}\right){\mathrm{e}}^{-\frac{1}{2}{\mathrm{x}}_{\mathrm{i}}^{2}})$	MS
F15	Shifted and rotated Rosenbrock	$\mathrm{f}(\mathrm{x})=\mathrm{SR}(\sum_{\mathrm{i}=1}^{\mathrm{n}-1}\left[100 {({\mathrm{x}}_{\mathrm{i}+1}- {\mathrm{x}}_{\mathrm{i}}^{2})}^{2} +{({\mathrm{x}}_{\mathrm{i}}-1)}^{2}\right])$	MN

**Table 3:** Some IEEE CEC functions applied to evaluate the IESOA method. Note: Shift is (S), and Shift Rotate (SR).

Basic function	Hybrid functions	Basic function	Hybrid functions
C1	S CEC01	C16	SR CEC14
C2	S CEC02	C17	S [CEC09, CEC08, CEC01]
C3	S CEC03	C18	S [CEC02, CEC12, CEC08]
C4	S CEC04	C19	S [CEC07, CEC06,CEC04, CEC14]
C5	S CEC05	C20	S [CEC12, CEC03,CEC13, CEC08]
C6	S CEC06	C21	S [CEC14, CEC12,CEC04, CEC09, CEC01]
C7	S CEC07	C22	S [CEC10, CEC11,CEC13, CEC09, CEC05]
C8	S CEC08	C23	S(1,2,3,4,5) [C04, C01,C02, C03, C01]
C9	SR CEC08	C24	S(1,2,3) [C10, C09,C14]
C10	S CEC09	C25	S(1,2,3) [C11, C09,C01]
C11	SR CEC09	C26	S(1,2,3,4,5) [C11,C13,C01,C06, C07]
C12	SR CEC10	C27	S(1,2,3,4,5) [C14,C09,C11,C06, C01]
C13	SR CEC11	C28	S(1,2,3,4,5) [C15,C13,C13,C11, C16, C1]
C14	SR CEC12	C29	S(4,5,6) [C17,C18,C19]
C15	SR CEC13	C30	S(1,2,3) [C20,C21,C22]

In addition to using classical benchmark functions, we also evaluated the performance of the IEOSA method using IEEE CEC functions listed in Table 3.

The outcome obtained from experimenting with the combined benchmark functions, consisting of classical and IEEE CEC functions, is reported and detailed in the next section.

## Results and discussion

After exhaustive experimentation of the methods proposed in this study, the results obtained are detailed in this section. The result presentation is sectioned into two major parts: firstly, the performance of the IEOSA metaheuristic algorithm, as evaluated with the classical benchmark functions and the IEEE CEC functions, and secondly, the performance of IEOSA with regard to its applicability in solving the feature reduction and minimization problem in medical images is evaluated and performance reported. Meanwhile, the performance evaluation follows the approach of investigating the convergence rate and the quality of solutions obtained, which were further compared with some state-of-the-art methods.

The performance evaluation of the proposed IEOSA was investigated using the listed benchmark functions. Exhaustive experimentation using 1000 epochs was applied for the training, while all algorithm-based parameters remained as described in “Methodology” section. In addition to experimenting with the benchmark functions on IEOSA, we also investigated the performance of the base algorithm, EOSA, to allow for comparative analysis. Furthermore, state-of-the-art optimization algorithms, which have been proposed in recent years, have also been experimented with under the same conditions, environmental setup, and benchmark functions. This is to support a fair comparative analysis of the proposed variant algorithm with the said algorithms, namely: Aquila Optimizer (AO), Arithmetic Optimization Algorithm (AOA), Archimedes Optimization Algorithm (ArchOA), Coronavirus Herd Immunity Optimization (CHIO), Genetic Algorithm (GA), Hunger Games Search (HGS), Invasive Weed Optimization (IWO), Sparrow Search Algorithm (SSA), Teaching Learning-Based Optimization (TLO), Virus Colony Search (VCS), and Wildebeest Herd Optimization (WHO). The algorithms were carefully selected from biology-based (IWO, VCS, and WHO), swarm-based (AO, HGS, and SSA), physics-based (ArchOA), evolutionary-based (GA), math-based (AOA), and human-based (TLO and CHIO) options.

The experimentation results showed that IEOSA competes well with all the state-of-the-art algorithms. In some instances, it performed better than the algorithms, while in others, it demonstrated a competitive performance. The total overall performance values were: AO (8), AOA (9), ArchOA (6), CHIO (1), GA (5), HGS (10), IWO (2), SSA (6), TLO (6), VCS (3), WHO (4), EOSA (2), and IEOSA (10), as seen in Table 4. Fifteen (15) constrained benchmark algorithms were investigated, and the resulting values were obtained. Among all the algorithms, we see HGS of the swarm-based type is strongly competitive with IEOSA, while the likes of AO and AOA trail behind this performance. IEOSA demonstrated superiority over ArchOA, CHIO, GA, HGS, IWO, SSA, TLO, VCS, and WHO algorithms. This result implies that the new variant performs better than the base algorithm, EOSA, and shows competitive performance with related algorithms which positions it as relevant in addressing optimization problems.

**Table 4:** comparison of the performance of AO, AOA, ArchOA, CHIO, GA, HGS, IWO, SSA, TLO, VCS, WHO, EOSA, and IEOSA using fifteen (15) benchmark functions.

F	Metric	AO	AOA	ArchOA	CHIO	GA	HGS	IWO	SSA	TLO	VCS	WHO	EOSA	IEOSA
F1	Best	0.00E+00	0.00E+00	1.54E−265	3.76E−02	3.05E−05	0.00E+00	1.65E−01	2.35E−82	2.09E−255	1.36E−05	1.59E−11	1.12E−06	0.00E+00
	Mean	4.96E−03	0.00E+00	6.35E−04	4.91E−02	3.61E−03	4.38E−06	1.65E−01	4.05E−06	3.63E−04	3.69E−03	1.16E−04	3.83E−02	2.94E−03
	Std	3.16E−02	0.00E+00	6.45E−03	3.53E−02	1.60E−02	1.07E−04	0.00E+00	1.28E−04	6.24E−03	5.01E−03	2.96E−03	6.65E−02	4.13E−02
	Worst	2.50E−01	0.00E+00	9.37E−02	4.25E−01	3.08E−01	3.31E−03	1.65E−01	4.05E−03	1.75E−01	1.67E−02	9.09E−02	1.81E−01	8.27E−01
	Median	0.00E+00	0.00E+00	2.62E−67	4.77E−02	1.70E−04	0.00E+00	1.65E−01	1.44E−47	9.35E−128	9.66E−04	2.60E−11	6.27E−05	0.00E+00
	Deviation	0.00E+00	0.00E+00	− 1.5E−265	− 3.76E−02	− 3.05E−05	0.00E+00	− 1.65E−01	− 2.35E−82	− 2.09E−255	− 1.36E−05	− 1.59E−11	− 1.42E−05	0.00E+00
F2	Best	1.00E+01	1.00E+01	1.00E+01	1.06E+01	1.00E+01	1.00E+01	1.00E+01	1.00E+01	1.00E+01	1.00E+01	1.00E+01	1.00E+01	1.00E+01
	Mean	1.16E+01	1.01E+01	1.01E+01	1.09E+01	1.01E+01	1.00E+01	1.04E+01	1.00E+01	1.00E+01	1.00E+01	1.00E+01	1.21E+01	1.81E+01
	Std	4.47E+00	7.28E−01	7.29E−01	6.76E−01	2.90E−01	2.60E−05	6.51E−01	9.50E−05	1.75E−01	1.40E−02	2.61E−01	2.53E+00	0.00E+00
	Worst	2.85E+01	2.50E+01	2.48E+01	1.99E+01	1.64E+01	1.00E+01	1.60E+01	1.00E+01	1.47E+01	1.01E+01	1.54E+01	1.78E+01	1.81E+01
	Median	1.00E+01	1.00E+01	1.00E+01	1.08E+01	1.00E+01	1.00E+01	1.00E+01	1.00E+01	1.00E+01	1.00E+01	1.00E+01	1.00E+01	1.81E+01
	Deviation	− 1.00E+01	− 1.00E+01	− 1.00E+01	− 1.06E+01	− 1.00E+01	− 1.00E+01	− 1.00E+01	− 1.00E+01	− 1.00E+01	− 1.00E+01	− 1.00E+01	− 1.00E+01	− 1.81E+01
F3	Best	1.32E−02	0.00E+00	0.00E+00	3.79E−01	1.92E+00	0.00E+00	3.21E−04	0.00E+00	0.00E+00	3.21E−08	0.00E+00	6.18E−02	0.00E+00
	Mean	2.15E+04	0.00E+00	1.94E+02	1.11E+04	1.68E+02	1.66E+02	1.17E+02	7.08E−02	7.17E+02	9.57E−03	4.02E−04	1.51E+06	5.58E+04
	Std	1.85E+05	0.00E+00	3.26E+03	1.91E+05	1.69E+03	3.06E+03	1.67E+03	2.24E+00	1.52E+04	1.41E−01	7.31E−03	2.24E+04	1.72E+06
	Worst	1.94E+06	0.00E+00	5.96E+04	3.49E+06	3.73E+04	7.74E+04	3.51E+04	7.08E+01	3.39E+05	2.24E+00	1.34E−01	2.01E+06	5.43E+07
	Median	1.16E+03	0.00E+00	7.49E−199	3.79E−01	5.27E+00	0.00E+00	2.14E−02	0.00E+00	3.95E−276	9.84E−06	0.00E+00	1.51E+06	0.00E+00
	Deviation	− 1.32E−02	0.00E+00	0.00E+00	− 3.79E−01	− 1.92E+00	0.00E+00	− 3.21E−04	0.00E+00	0.00E+00	− 3.21E−08	0.00E+00	− 1.51E+06	0.00E+00
F4	Best	7.15E−06	4.00E−01	1.11E−21	5.53E−02	1.12E−04	3.55E−06	3.27E−01	1.35E−32	1.35E−32	5.86E−02	1.55E−06	1.26E−01	8.70E+00
	Mean	5.73E−03	4.00E−01	2.27E−03	8.47E−02	8.61E−03	2.01E−03	3.28E−01	4.25E−05	1.69E−03	6.08E−02	2.14E−04	2.19E−01	8.70E+00
	Std	6.44E−02	5.55E−17	2.61E−02	9.22E−02	2.06E−02	2.14E−02	9.43E−03	1.34E−03	2.70E−02	1.41E−02	3.00E−03	1.48E−01	0.00E+00
	Worst	1.00E+00	4.00E−01	5.44E−01	7.04E−01	3.05E−01	5.69E−01	6.26E−01	4.24E−02	6.15E−01	2.65E−01	8.61E−02	6.52E−01	8.70E+00
	Median	1.61E−03	4.00E−01	1.11E−21	5.53E−02	1.41E−04	3.55E−06	3.27E−01	1.35E−32	1.35E−32	5.86E−02	1.64E−06	1.27E−01	8.70E+00
	Deviation	− 7.15E−06	− 4.00E−01	− 1.11E−21	− 5.53E−02	− 1.12E−04	− 3.55E−06	− 3.27E−01	− 1.35E−32	− 1.35E−32	− 5.86E−02	− 1.55E−06	− 1.26E−01	− 8.70E+00
F5	Best	4.50E+00	4.50E+00	4.53E+00	4.59E+00	4.50E+00	4.50E+00	4.63E+00	4.50E+00	4.50E+00	4.50E+00	4.53E+00	4.50E+00	4.50E+00
	Mean	4.66E+00	4.50E+00	4.53E+00	4.59E+00	4.51E+00	4.50E+00	4.63E+00	4.50E+00	4.50E+00	4.52E+00	4.55E+00	4.57E+00	4.50E+00
	Std	8.93E−02	0.00E+00	9.91E−03	2.47E−02	1.13E−02	6.15E−03	8.88E−16	7.66E−03	7.16E−03	1.89E−02	3.65E−02	9.47E−02	2.22E−02
	Worst	4.85E+00	4.50E+00	4.66E+00	4.78E+00	4.74E+00	4.66E+00	4.63E+00	4.74E+00	4.58E+00	4.68E+00	4.70E+00	4.81E+00	4.80E+00
	Median	4.71E+00	4.50E+00	4.53E+00	4.59E+00	4.51E+00	4.50E+00	4.63E+00	4.50E+00	4.50E+00	4.50E+00	4.53E+00	4.50E+00	4.50E+00
	Deviation	− 4.50E+00	− 4.50E+00	− 4.53E+00	− 4.59E+00	− 4.50E+00	− 4.50E+00	− 4.63E+00	− 4.50E+00	− 4.50E+00	− 4.50E+00	− 4.53E+00	− 4.50E+00	− 4.50E+00
F6	Best	9.39E−05	1.61E−05	3.71E−04	1.47E−02	2.24E−03	5.96E−06	1.05E−01	3.80E−05	1.12E−04	9.83E−05	5.76E−05	3.86E−03	2.64E−06
	Mean	1.16E−02	9.67E−05	2.89E−03	2.20E−02	2.92E−03	4.04E−04	1.07E−01	3.85E−04	1.66E−03	4.38E−03	4.93E−04	7.35E−02	7.07E+00
	Std	6.31E−02	9.28E−04	2.72E−02	3.63E−02	1.21E−02	1.71E−03	9.24E−03	2.46E−03	1.48E−02	7.34E−03	4.49E−03	1.43E−01	1.36E−02
	Worst	4.87E−01	2.84E−02	4.84E−01	4.48E−01	2.80E−01	1.51E−02	2.94E−01	7.43E−02	3.44E−01	4.90E−02	1.31E−01	7.74E−01	7.16E+00
	Median	1.32E−04	1.97E−05	7.19E−04	1.47E−02	2.24E−03	1.00E−04	1.05E−01	9.45E−05	1.12E−04	1.47E−03	5.76E−05	7.55E−03	7.07E+00
	Deviation	− 9.39E−05	− 1.61E−05	− 3.71E−04	− 1.47E−02	− 2.24E−03	− 5.96E−06	− 1.05E−01	− 3.80E−05	− 1.12E−04	− 9.83E−05	− 5.76E−05	− 3.86E−03	− 7.06E+00
F7	Best	− 5.26E+01	− 3.05E+01	− 4.56E+01	− 5.13E+01	− 5.29E+01	− 5.08E+01	− 4.87E+01	− 5.28E+01	− 5.26E+01	− 4.80E+01	− 5.24E+01	− 5.25E+01	− 4.75E+01
	Mean	− 5.08E+01	− 3.05E+01	− 4.56E+01	− 4.97E+01	− 5.26E+01	− 5.06E+01	− 4.84E+01	− 5.26E+01	− 5.05E+01	− 4.78E+01	− 5.15E+01	− 4.19E+01	− 4.44E+01
	Std	1.80E+00	0.00E+00	3.11E−01	1.56E+00	5.37E−01	9.23E−01	1.40E+00	1.54E+00	1.92E+00	1.06E+00	1.58E+00	3.53E+00	2.36E+00
	Worst	− 3.85E+01	− 3.05E+01	− 4.31E+01	− 4.36E+01	− 4.47E+01	− 3.74E+01	− 4.07E+01	− 3.84E+01	− 4.10E+01	− 3.52E+01	− 4.16E+01	− 3.59E+01	− 1.77E+01
	Median	− 5.04E+01	− 3.05E+01	− 4.56E+01	− 4.95E+01	− 5.28E+01	− 5.08E+01	− 4.87E+01	− 5.28E+01	− 5.07E+01	− 4.80E+01	− 5.18E+01	− 4.29E+01	− 4.46E+01
	Deviation	5.26E+01	3.05E+01	4.56E+01	5.13E+01	5.29E+01	5.08E+01	4.87E+01	5.28E+01	5.26E+01	4.80E+01	5.24E+01	4.58E+01	4.75E+01
F8	Best	0.00E+00	0.00E+00	0.00E+00	2.81E−01	7.32E−03	0.00E+00	4.82E+02	1.32E−14	7.76E−09	1.20E−06	2.88E−09	3.02E−02	0.00E+00
	Mean	3.66E+00	6.18E−04	1.04E−01	1.31E+00	6.97E−02	5.41E−02	6.56E+02	2.48E−05	2.04E−02	1.02E−03	2.08E−03	4.00E−01	3.38E+02
	Std	1.11E+01	1.46E−02	1.85E+00	4.25E+00	4.39E−01	9.42E−01	2.35E+02	3.38E−04	3.65E−01	7.48E−03	4.65E−02	3.32E−01	5.68E−14
	Worst	8.77E+01	4.31E−01	4.08E+01	6.27E+01	1.21E+01	2.22E+01	1.12E+03	6.18E−03	1.04E+01	1.62E−01	1.35E+00	1.27E+00	3.38E+02
	Median	1.91E−234	0.00E+00	0.00E+00	7.41E−01	7.62E−03	0.00E+00	4.82E+02	1.32E−14	1.66E−07	2.14E−04	6.58E−09	5.02E−01	3.38E+02
	Deviation	0.00E+00	0.00E+00	0.00E+00	− 2.81E−01	− 7.32E−03	0.00E+00	− 4.82E+02	− 1.32E−14	− 7.76E−09	− 1.20E−06	− 2.88E−09	− 3.02E−02	− 3.38E+02
F9	Best	2.28E+00	0.00E+00	0.00E+00	1.42E+01	7.86E−02	0.00E+00	− 1.46E+03	1.78E−15	0.00E+00	6.22E−03	3.26E+00	1.71E+01	0.00E+00
	Mean	1.17E+01	0.00E+00	1.12E+00	1.67E+01	1.52E+00	3.17E−02	− 1.46E+03	7.09E−03	5.19E−01	7.64E−01	3.75E+00	2.10E+01	2.01E−01
	Std	6.35E+00	0.00E+00	3.52E+00	4.68E+00	1.97E+00	9.97E−01	2.27E−13	2.23E−01	2.29E+00	1.01E+00	1.08E+00	2.67E+00	2.47E+00
	Worst	3.09E+01	0.00E+00	2.63E+01	5.18E+01	2.32E+01	3.15E+01	− 1.46E+03	7.04E+00	4.38E+01	8.17E+00	3.06E+01	2.68E+01	4.62E+01
	Median	1.15E+01	0.00E+00	0.00E+00	1.42E+01	1.10E−01	0.00E+00	− 1.46E+03	1.78E−15	0.00E+00	6.07E−01	3.58E+00	2.19E+01	0.00E+00
	Deviation	− 2.28E+00	0.00E+00	0.00E+00	− 1.42E+01	− 7.86E−02	0.00E+00	1.04E+03	− 1.78E−15	0.00E+00	− 6.22E−03	− 3.26E+00	− 1.71E+01	0.00E+00
F10	Best	0.00E+00	0.00E+00	4.65E−104	4.65E+01	2.02E+01	0.00E+00	1.56E+03	1.12E−16	4.26E−197	2.20E−06	1.14E−06	4.06E+02	0.00E+00
	Mean	2.66E+01	0.00E+00	8.55E+00	1.51E+02	6.63E+01	4.64E+00	1.56E+03	2.79E−06	4.76E−01	1.42E−03	6.96E−01	6.34E+02	1.24E+04
	Std	1.25E+02	0.00E+00	6.25E+01	4.14E+02	8.79E+01	9.91E+01	4.55E−13	6.16E−05	8.08E+00	2.44E−02	1.89E+01	7.49E+01	1.13E+03
	Worst	2.17E+03	0.00E+00	8.86E+02	2.80E+03	2.08E+03	2.24E+03	1.56E+03	1.38E−03	2.39E+02	7.71E−01	5.91E+02	9.00E+02	4.82E+04
	Median	0.00E+00	0.00E+00	1.31E−44	4.65E+01	3.45E+01	0.00E+00	1.56E+03	1.75E−12	6.18E−97	5.02E−04	1.28E−06	6.13E+02	1.23E+04
	Deviation	0.00E+00	0.00E+00	− 4.65E−104	− 4.65E+01	− 2.02E+01	0.00E+00	− 1.56E+03	− 1.12E−16	− 4.26E−197	− 2.20E−06	− 1.14E−06	− 6.13E+02	− 1.23E+04
F11	Best	− 1.66E+03	− 1.05E+03	− 1.28E+03	− 1.72E+03	− 2.09E+03	− 1.46E+03	3.28E+01	− 1.02E+03	− 1.98E+03	− 7.46E+02	− 1.34E+03	− 1.17E+03	− 9.03E+02
	Mean	− 1.60E+03	− 1.05E+03	− 1.27E+03	− 1.63E+03	− 2.07E+03	− 1.46E+03	3.28E+01	− 1.02E+03	− 1.95E+03	− 7.46E+02	− 1.34E+03	− 1.05E+03	− 1.13E+03
	Std	8.64E+01	2.27E−13	3.91E+00	1.58E+02	5.67E+01	1.14E+01	7.11E−15	7.08E+00	7.71E+01	2.27E−13	5.05E+00	1.05E+02	1.02E+01
	Worst	− 9.19E+02	− 1.05E+03	− 1.27E+03	− 8.71E+02	− 1.06E+03	− 1.33E+03	3.28E+01	− 8.88E+02	− 1.25E+03	− 7.46E+02	− 1.19E+03	− 7.74E+02	− 9.03E+02
	Median	− 1.63E+03	− 1.05E+03	− 1.28E+03	− 1.72E+03	− 2.09E+03	− 1.46E+03	3.28E+01	− 1.02E+03	− 1.98E+03	− 7.46E+02	− 1.34E+03	− 9.71E+02	− 1.13E+03
	Deviation	1.24E+03	6.29E+02	8.58E+02	1.30E+03	1.68E+03	1.04E+03	− 3.28E+01	6.04E+02	1.56E+03	3.27E+02	9.21E+02	7.56E+02	7.13E+02
F12	Best	0.00E+00	0.00E+00	6.04E−112	8.13E+01	6.60E−01	0.00E+00	1.03E+04	2.17E−79	6.75E−250	1.69E−05	1.15E−07	3.83E+02	0.00E+00
	Mean	3.12E+01	0.00E+00	1.93E+01	3.47E+02	1.56E+01	8.03E−02	1.76E+04	1.02E−06	4.52E+00	1.38E−02	7.83E−01	6.49E+03	7.51E+00
	Std	1.34E+02	0.00E+00	1.31E+02	4.14E+02	8.87E+01	1.51E+00	1.76E+04	2.04E−05	7.22E+01	3.39E−01	2.00E+01	1.08E+03	1.99E+02
	Worst	2.08E+03	0.00E+00	2.18E+03	3.72E+03	2.59E+03	3.81E+01	6.52E+04	4.51E−04	1.86E+03	1.07E+01	6.12E+02	7.61E+03	6.29E+03
	Median	0.00E+00	0.00E+00	1.86E−49	3.12E+02	7.86E−01	0.00E+00	1.03E+04	2.49E−45	1.11E−123	3.07E−03	8.20E−07	6.17E+03	0.00E+00
	Deviation	0.00E+00	0.00E+00	− 6.04E−112	− 8.13E+01	− 6.60E−01	0.00E+00	− 1.03E+04	− 2.17E−79	− 6.75E−250	− 1.69E−05	− 1.15E−07	− 3.83E+03	0.00E+00
F13	Best	1.20E+03	1.20E+03	1.20E+03	1.20E+03	1.20E+03	1.20E+03	9.23E+02	1.20E+03	1.20E+03	1.20E+03	1.20E+03	1.21E+03	1.20E+03
	Mean	1.20E+03	1.20E+03	1.20E+03	1.20E+03	1.20E+03	1.20E+03	9.23E+02	1.20E+03	1.20E+03	1.20E+03	1.20E+03	1.21E+03	2.19E+03
	Std	5.53E−01	2.27E−13	2.57E−01	3.87E−01	1.87E−01	5.79E−02	5.89E−01	9.88E−05	1.62E−01	2.12E−03	6.57E−02	1.78E−02	4.55E−13
	Worst	1.21E+03	1.20E+03	1.20E+03	1.20E+03	1.20E+03	1.20E+03	9.42E+02	1.20E+03	1.20E+03	1.20E+03	1.20E+03	1.21E+03	2.19E+03
	Median	1.20E+03	1.20E+03	1.20E+03	1.20E+03	1.20E+03	1.20E+03	9.23E+02	1.20E+03	1.20E+03	1.20E+03	1.20E+03	1.21E+03	2.19E+03
	Deviation	− 1.20E+03	− 1.20E+03	− 1.20E+03	− 1.20E+03	− 1.20E+03	− 1.20E+03	− 9.23E+02	− 1.20E+03	− 1.20E+03	− 1.20E+03	− 1.20E+03	− 1.21E+03	− 2.19E+03
F14	Best	1.20E+03	1.21E+03	1.20E+03	1.21E+03	1.20E+03	1.20E+03	5.88E+03	1.20E+03	1.20E+03	1.23E+03	1.20E+03	1.21E+03	1.21E+03
	Mean	1.21E+03	1.21E+03	1.20E+03	1.21E+03	1.21E+03	1.20E+03	5.88E+03	1.21E+03	1.20E+03	1.23E+03	1.20E+03	1.22E+03	3.06E+04
	Std	1.02E+00	0.00E+00	1.12E+00	9.56E−01	2.99E+00	5.19E−01	1.85E+00	4.95E+00	3.38E−01	6.82E−13	8.58E−02	1.03E+00	1.79E+05
	Worst	1.21E+03	1.21E+03	1.23E+03	1.21E+03	1.23E+03	1.22E+03	5.89E+03	1.24E+03	1.21E+03	1.23E+03	1.21E+03	1.22E+03	5.68E+06
	Median	1.21E+03	1.21E+03	1.20E+03	1.21E+03	1.20E+03	1.20E+03	5.88E+03	1.20E+03	1.20E+03	1.23E+03	1.20E+03	1.22E+03	2.49E+04
	Deviation	− 1.20E+03	− 1.21E+03	− 1.20E+03	− 1.21E+03	− 1.20E+03	− 1.20E+03	− 5.88E+03	− 1.20E+03	− 1.20E+03	− 1.23E+03	− 1.20E+03	− 1.22E+03	− 2.49E+04
F15	Best	4.00E+02	4.01E+02	4.00E+02	4.07E+02	4.03E+02	4.00E+02	1.06E−01	4.00E+02	4.00E+02	4.00E+02	4.00E+02	4.81E+02	4.54E+02
	Mean	4.05E+02	4.01E+02	4.08E+02	4.12E+02	4.04E+02	4.00E+02	1.14E−01	4.00E+02	4.00E+02	4.00E+02	4.00E+02	5.42E+02	6.41E+03
	Std	2.55E+01	1.71E−13	2.39E+01	9.38E+00	3.86E+00	9.43E−01	1.22E−02	1.18E−04	4.49E+00	1.14E−01	2.42E+00	6.88E+01	9.09E−13
	Worst	6.23E+02	4.01E+02	7.02E+02	4.54E+02	4.60E+02	4.24E+02	1.59E−01	4.00E+02	4.93E+02	4.04E+02	4.71E+02	6.36E+02	6.41E+03
	Median	4.00E+02	4.01E+02	4.00E+02	4.07E+02	4.03E+02	4.00E+02	1.06E−01	4.00E+02	4.00E+02	4.00E+02	4.00E+02	4.87E+02	6.41E+03
	Deviation	− 4.00E+02	− 4.01E+02	− 4.00E+02	− 4.07E+02	− 4.03E+02	− 4.00E+02	− 1.06E−01	− 4.00E+02	− 4.00E+02	− 4.00E+02	− 4.00E+02	− 4.81E+02	− 6.41E+03
Total		8	9	6	1	5	10	2	6	6	3	4	2	10

The swarm-based algorithms are famously known to yield strong results and performance in optimization problems. Interestingly, the proposed variant of EOSA, namely IEOSA, has demonstrated an extremely competitive performance compared with the swarm-based algorithms. For instance, IEOSA achieved a parallel performance with HGS of the swarm intelligence category, outperformed AO by 10 versus 8, and outperformed SSA by 10 versus 6. Note that both AO and SSA are swarm based. This further demonstrates the applicability of this new variant, IEOSA, to solve most of the critical optimization problems which swarm-based methods have been reputed to address. Furthermore, we discovered that the proposed new variant also outperformed the classical GA, since the IEOSA has 10 scores as against the 5 which is reported for GA. Interestingly, a comparison of the new variant algorithm with a recent and well-performing algorithm, AOA, has shown that IEOSA is strongly relevant and useful in addressing optimization problems, with better performance than the AOA. This argument is based on the fact that AOA reported 9 scores while IEOSA reported 10 scores.

A similar trend is observed for the physics-based and human-based algorithms. ArchOA, TLO, and CHIO reported 6, 6, and 1 scores, respectively, while IEOSA reported 10. A parallel comparison of IEOSA with those in its biology-based category showed that IEOSA outperformed them all, because IWO, VCS, and WHO yielded only 2, 3, and 4 scores for all 15 functions evaluated. In contrast, IEOSA showed good performance for ten benchmark functions. These results imply that IEOSA, a new variant of the EOSA algorithm, is a strong and highly competitive metaheuristic algorithm which should be investigated in solving optimization problems. This further reveals findings that all optimization problems which SSA, AOA, AO, TLO, CHIO, ArchOA, IWO, VCS and WHO have adequately solved, can also be conveniently solved using IEOSA, since these performance evaluations with benchmark functions have revealed that it is robust and fit for such. Interestingly, even where IEOSA is outperformed by other methods, as in the case of F4, F7, and F14, the differences are minimal, showing that the limitation of IEOSA is minimal and does not translate into any significant performance reduction.

**Figure 6:**
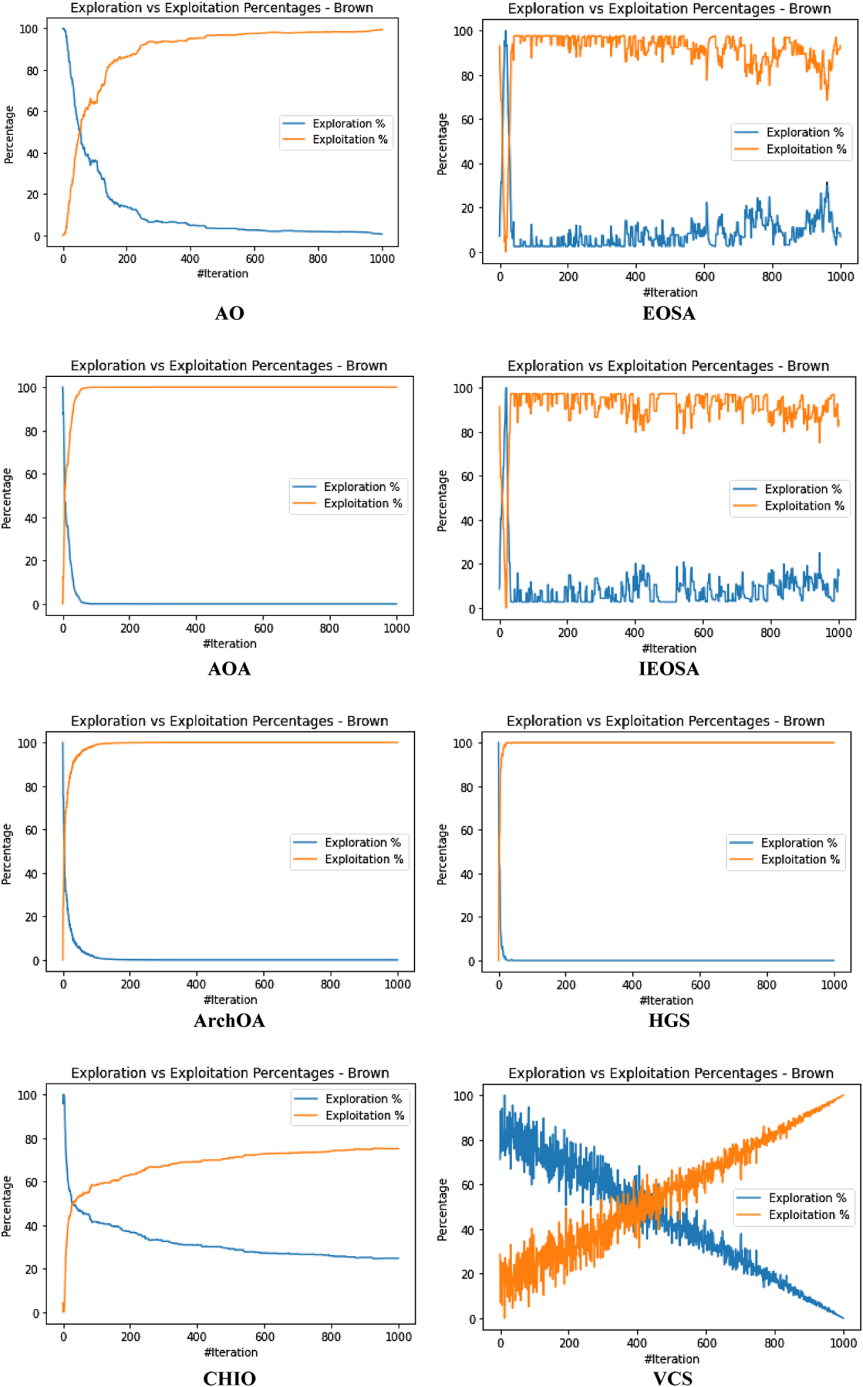

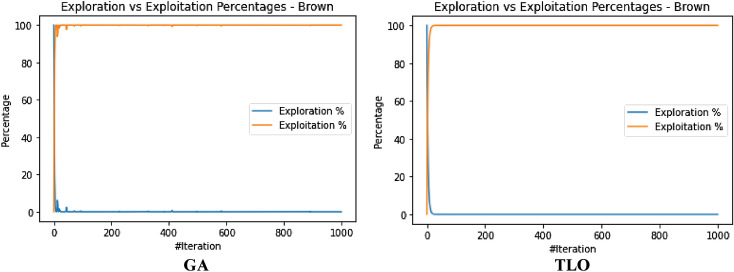
Illustration of the exploration and exploitation of Brown (F2) function for IEOSA as compared to AO, EOSA, AOA, IEOSA, ArchOA, HGS, CHIO, VCS, GA, and TLO.

The patterns of exploration and exploitation for all algorithms experimented with were captured and graphed, as shown in Fig. 6. Most algorithms demonstrated a good curve pattern in navigating their way during the search process for both local and global searches. For instance, we see the search curves for AO, AOA, HGS, CHIO, VCS, GA, TLO, EOSA, and IEOSA follow a pattern relevant to literature about local and global searches. In most cases, the global search for the algorithms began at 0 and peaked at the upper bound, while the local search dropped from the upper bound to 0 starting from epoch 1 to 1,000. Only VCS demonstrated poor performance in relation to the exploration and exploitation curves. The search curves for EOSA and IEOSA showed some staggering patterns while searching for the best solutions but maintained the fundamental pattern demonstrated by others. The implication of this search process at the intensification and diversification showed that IEOSA cannot be trapped into a local optimum, but can search for the best solution within the search space.

**Figure 7:**
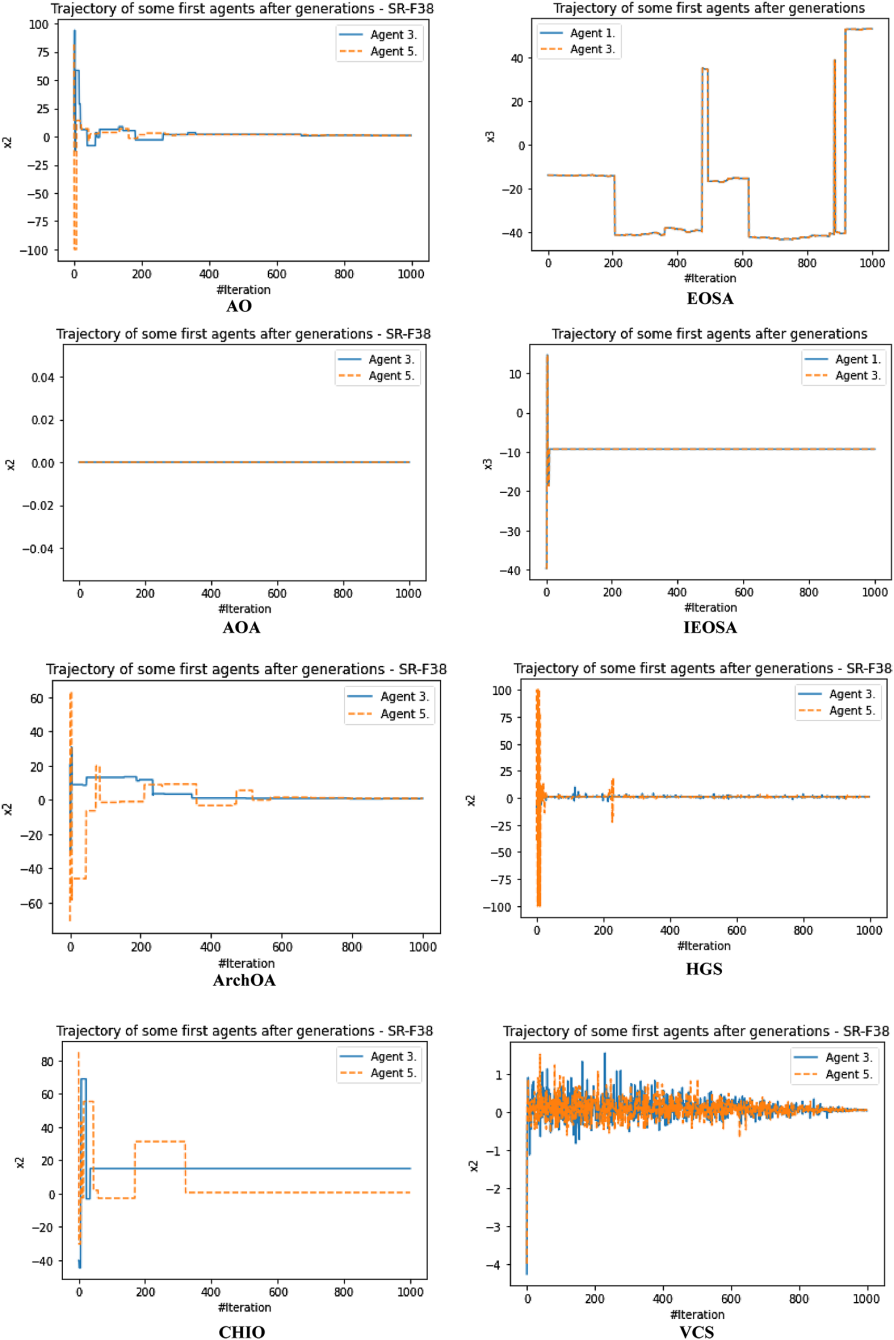

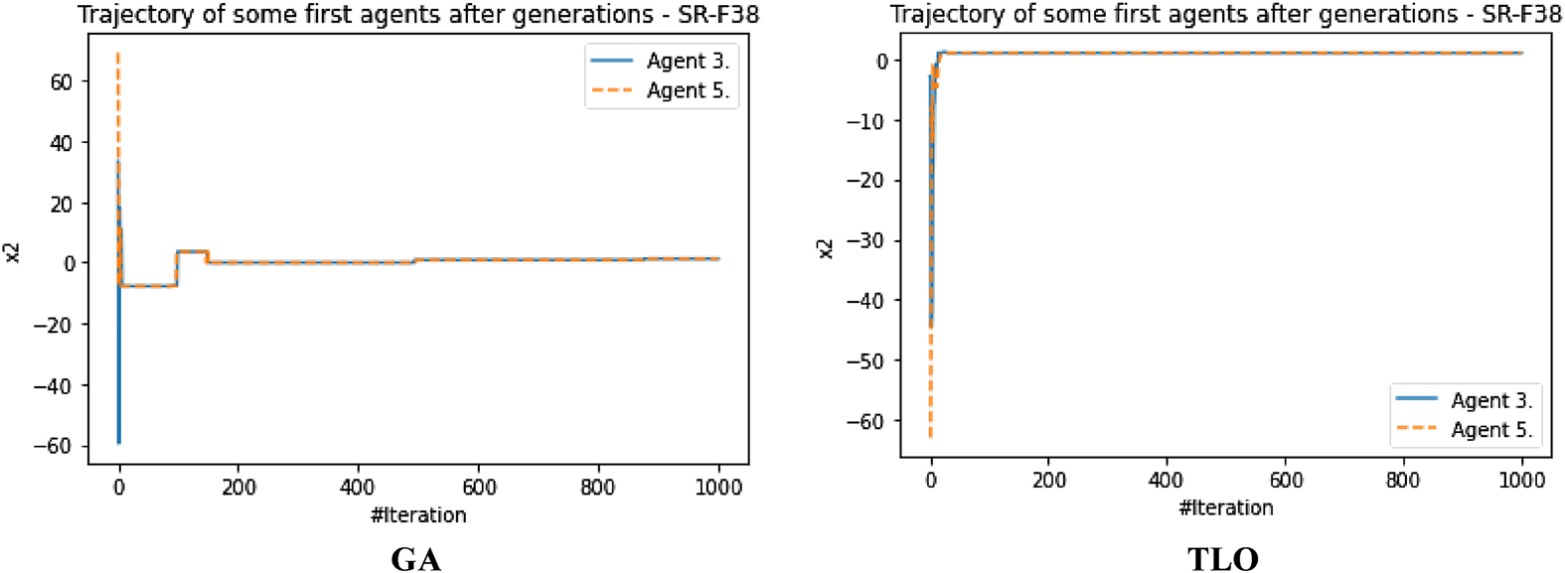
A comparative analysis of the trajectory for Shift-Rotated (SR) of Sum of Different Power (F13) for AO, EOSA, AOA, IEOSA, ArchOA, HGS, CHIO, VCS, GA, and TLO.

The curve of the trajectory of optimization of some solutions randomly selected is shown in Fig. 7. During the random selection, agent/solutions 3 and 5 were picked for graphing to investigate how each algorithm can optimize the solutions in the population during all training processes. Results obtained and graphed showed that IEOSA performed well in its optimization process for the two agents. This follows a similar trend in those of the competitive algorithms, although ArchOA, VCS, and CHIO failed to report good performance. The curve for the base algorithm showed a good and uniform optimization of all individuals, though with some staggering patterns.

**Figure 8:**
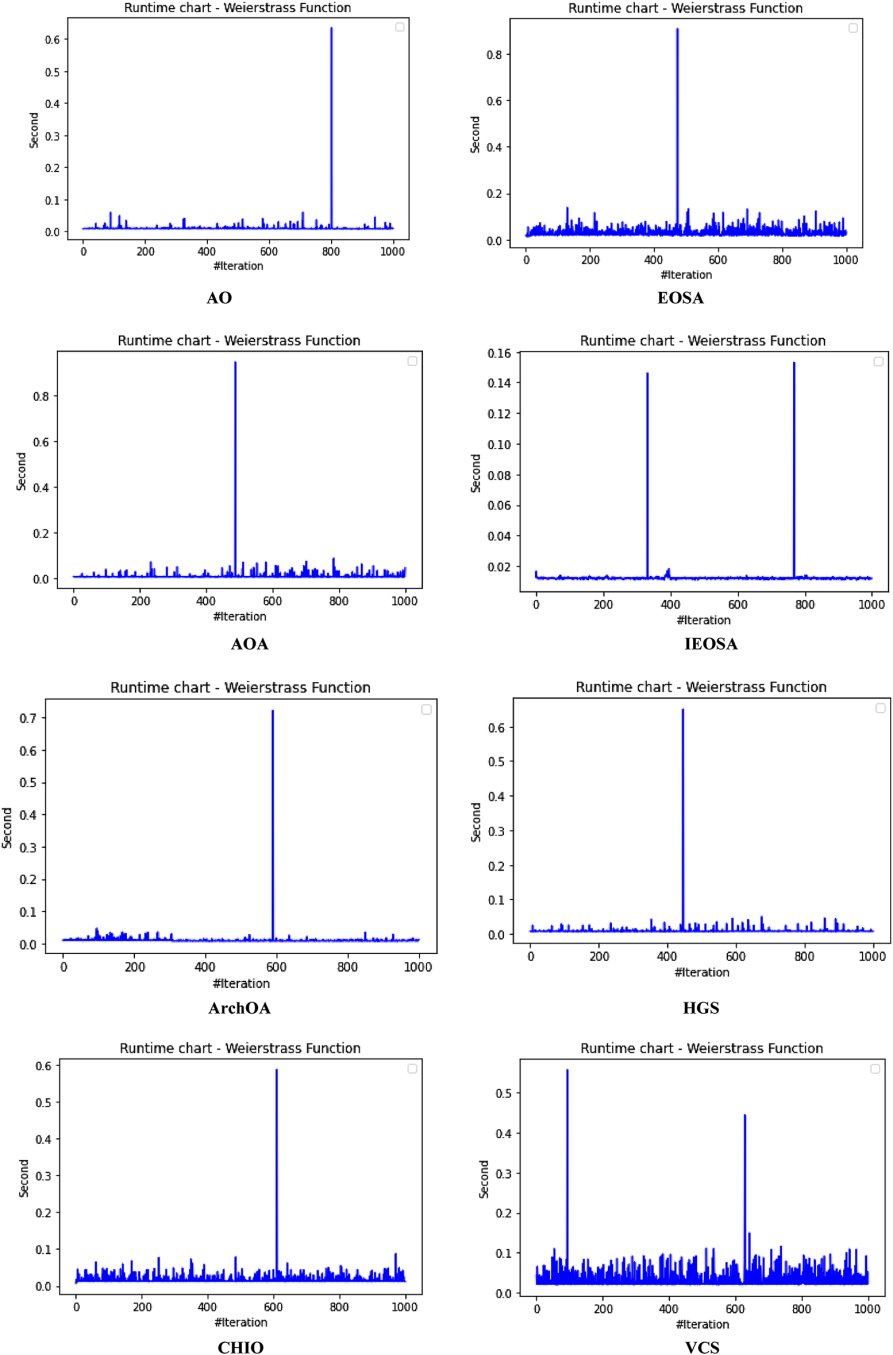

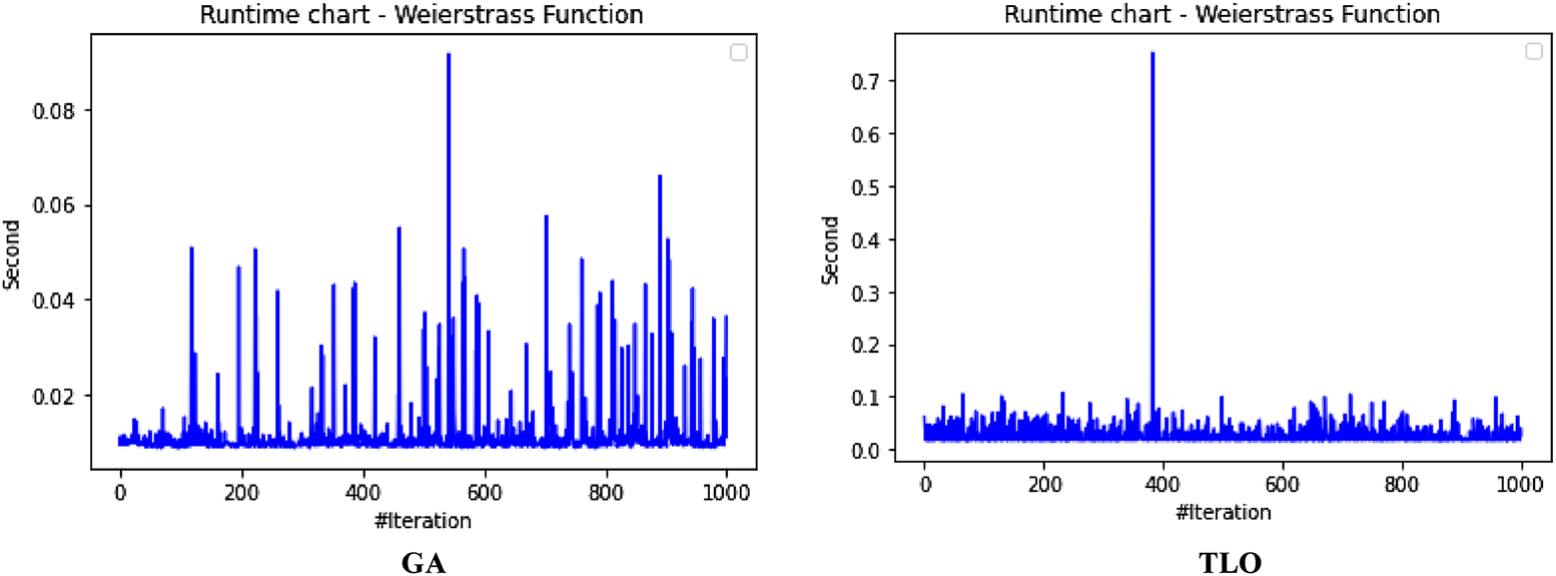
Performance evaluation of the runtime in running generalized penalized function (F4) for IEOSA, comparatively with AO, EOSA, AOA, IEOSA, ArchOA, HGS, CHIO, VCS, GA, and TLO.

The runtime of the algorithms was also calibrated, and the result obtained was graphed for discussion, as seen in Fig. 8. The GA, CHIO, VCS, TLO, and EOSA appear to show some measure of computational expense over other algorithms. This graphing is important to show what computational cost is required for the optimization process of each algorithm. Interestingly, we see the new variant optimization algorithm, IEOSA, showing competitive computational cost compared with other algorithms whose solutions also competed with it. This, therefore, implies that the proposed algorithm can provide an optimization solution to the optimization problem in a very cost-effective manner. This is very relevant to the application domain being considered in this study. The proposed optimization algorithm can solve the feature selection and reduction optimization problem in medical imaging with minimal computational cost. Results obtained for the runtime of the IEOSA when compared with other related methods showed that the runtime for the algorithm is far lower than those of AO, AOA, ArchOA, CHIO, GA, HGS, IWO, SSA, TLO, VCS, and WHO. As an example, where IEOSA obtained 0.16 as the computational running cost, the cost for the same function for other algorithms was AO (0.6), AOA (0.8), ArchOA (0.7), HGS (0.6), TLO (0.7), and CHIO (0.6).

**Figure 9:**
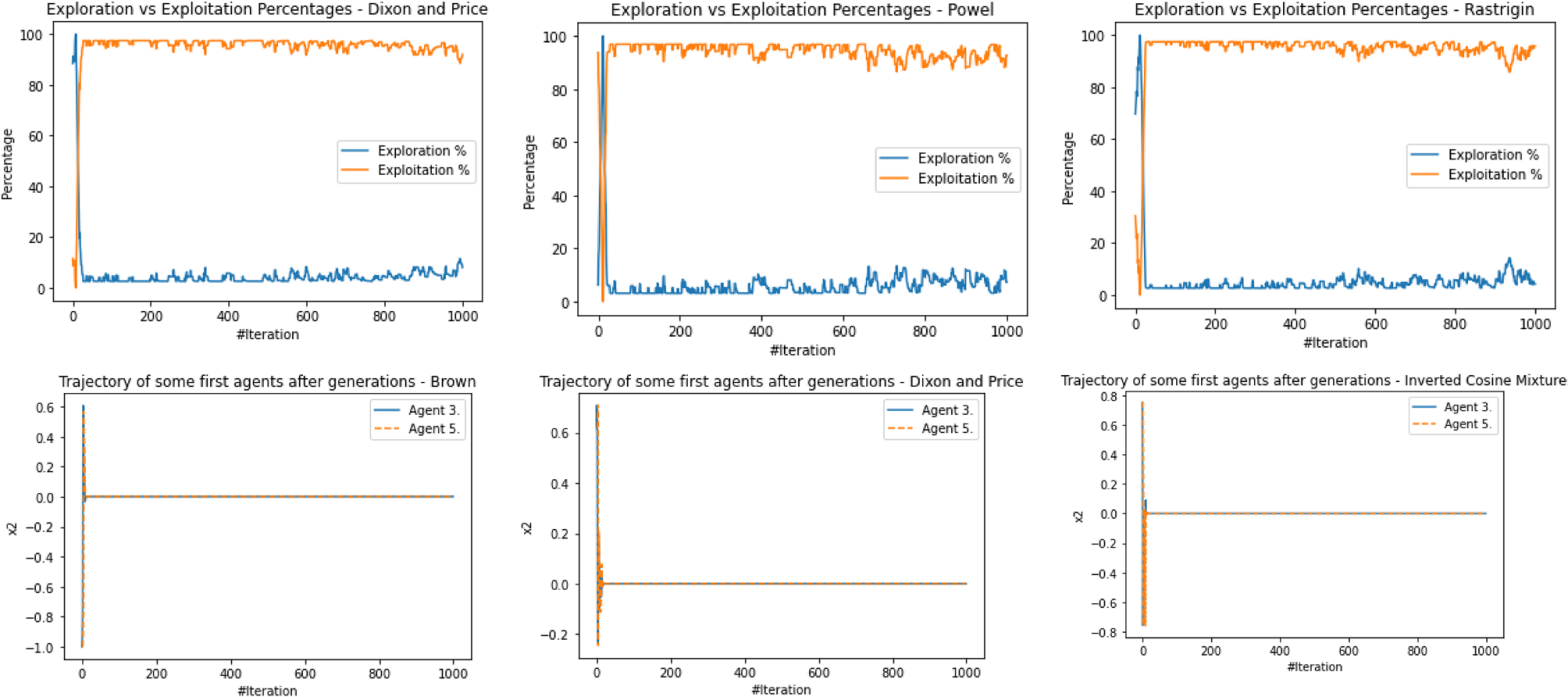
A graphical illustration of the convergence of IEOSA based on the trajectory of some selected solutions for Brown, Dixon and Price and Inverted Cosine Mixture functions, and the overall exploration and exploitation of Dixon and Price, Powel, and Rastrigen functions.

Further to the comparative performance of IEOSA discussed in previous paragraphs, the graph plots for some selected benchmark functions on the curve for exploration and exploitation and trajectory for solution optimization are shown in Fig. 9. These plots further confirm the algorithm’s effectiveness, which showed a very smooth curve for Brown, Dixon and Price, and Inverted Cosine Mixture benchmark functions.

**Figure 10:**
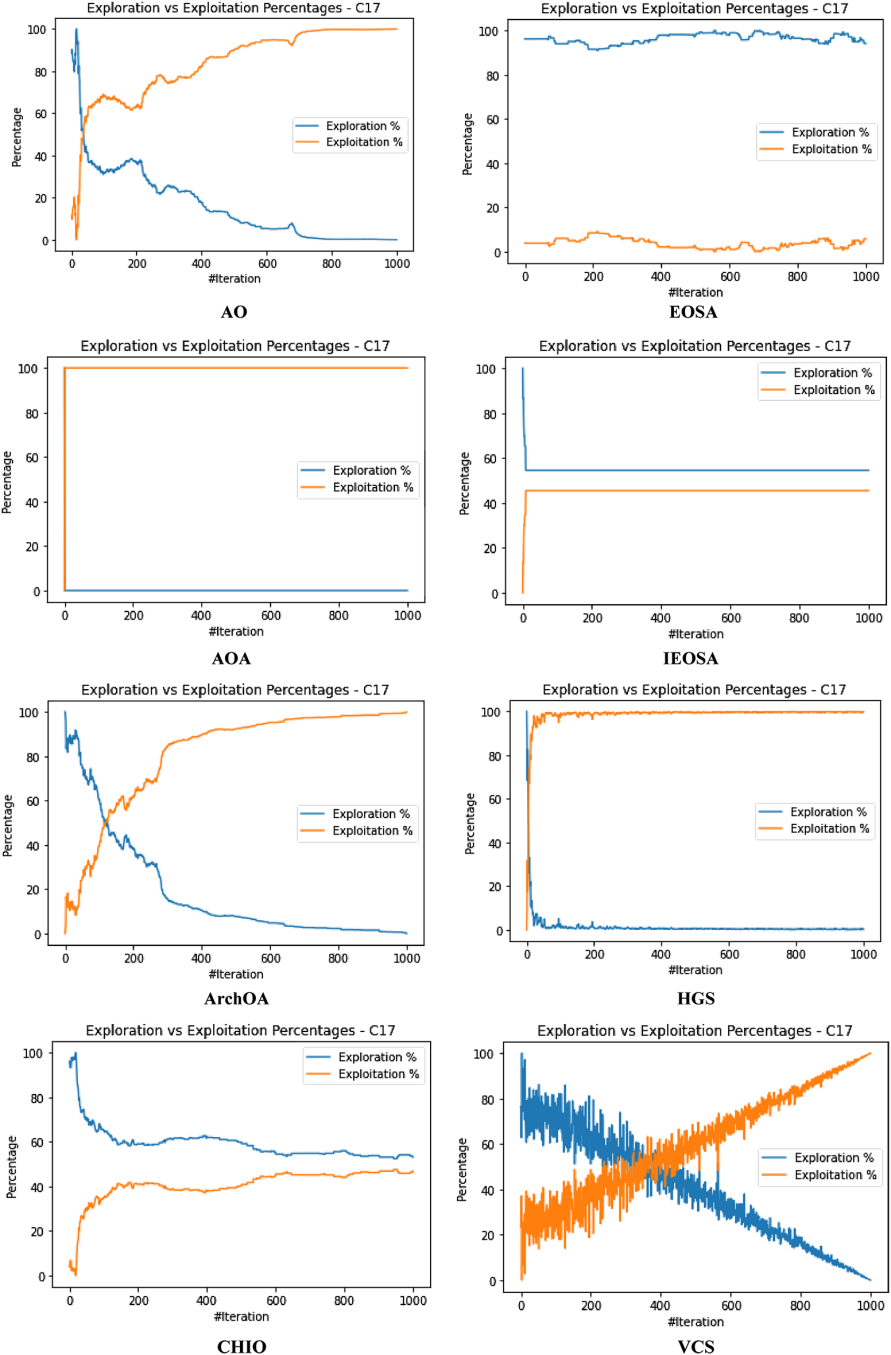

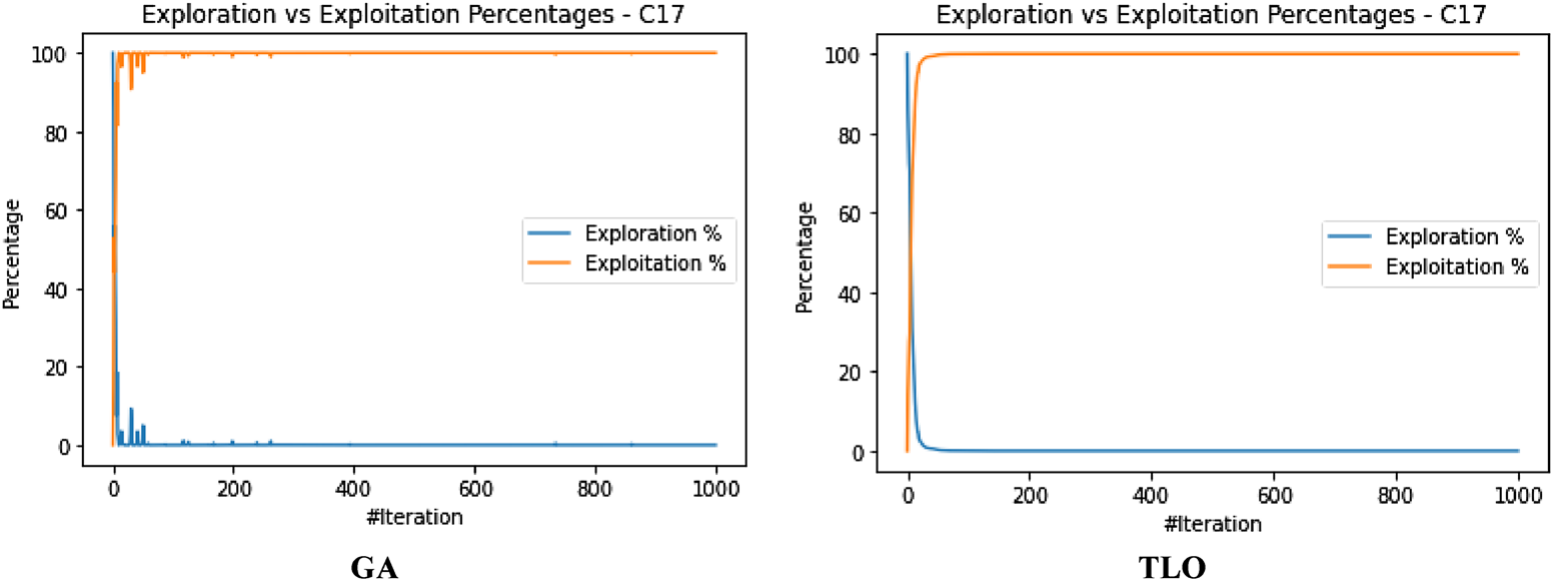
Illustration of the exploration and exploitation of Brown (F2) function for IEOSA as compared to AO, EOSA, AOA, ArchOA, HGS, CHIO, VCS, GA, and TLO.

**Figure 11:**
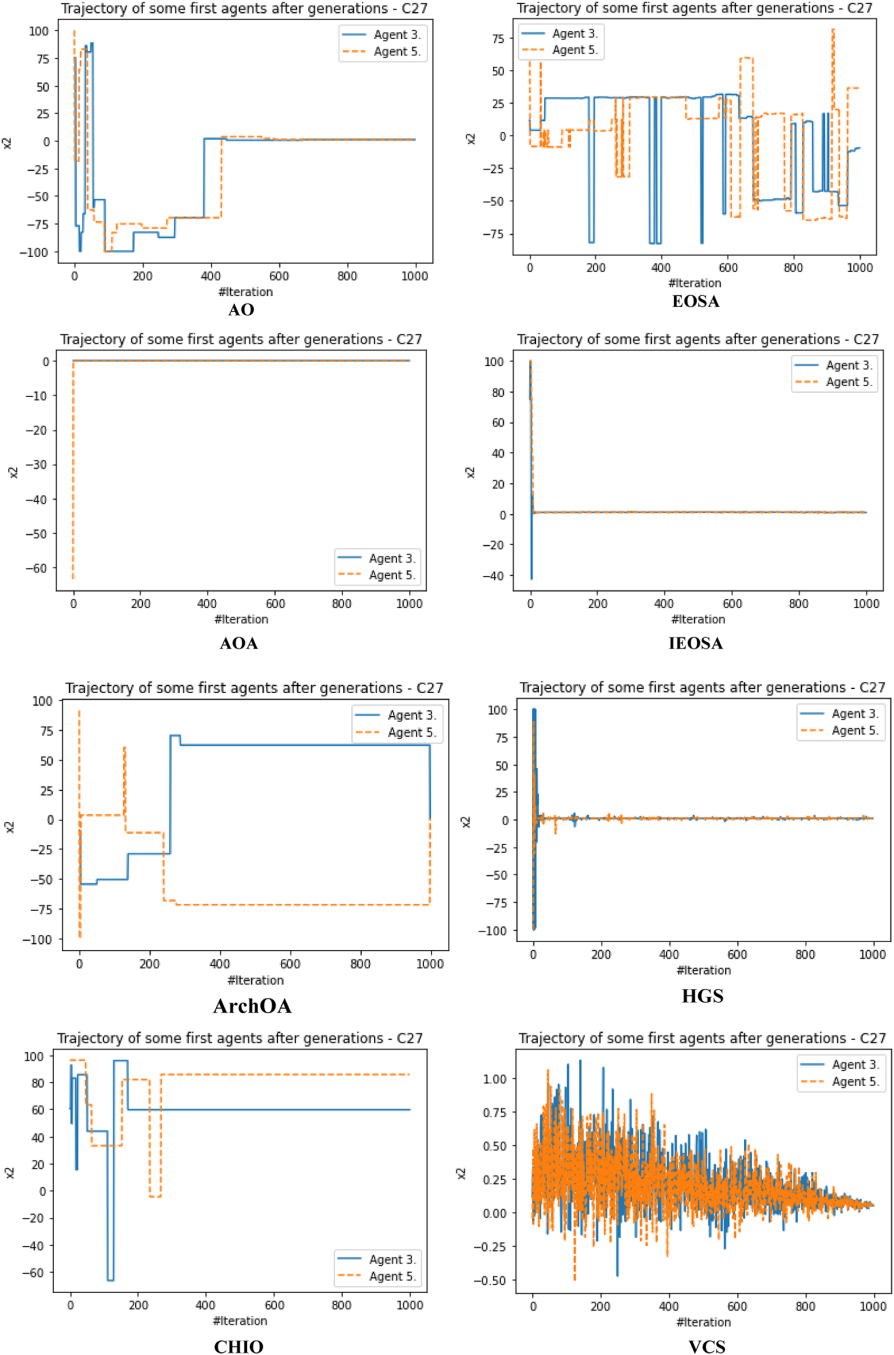

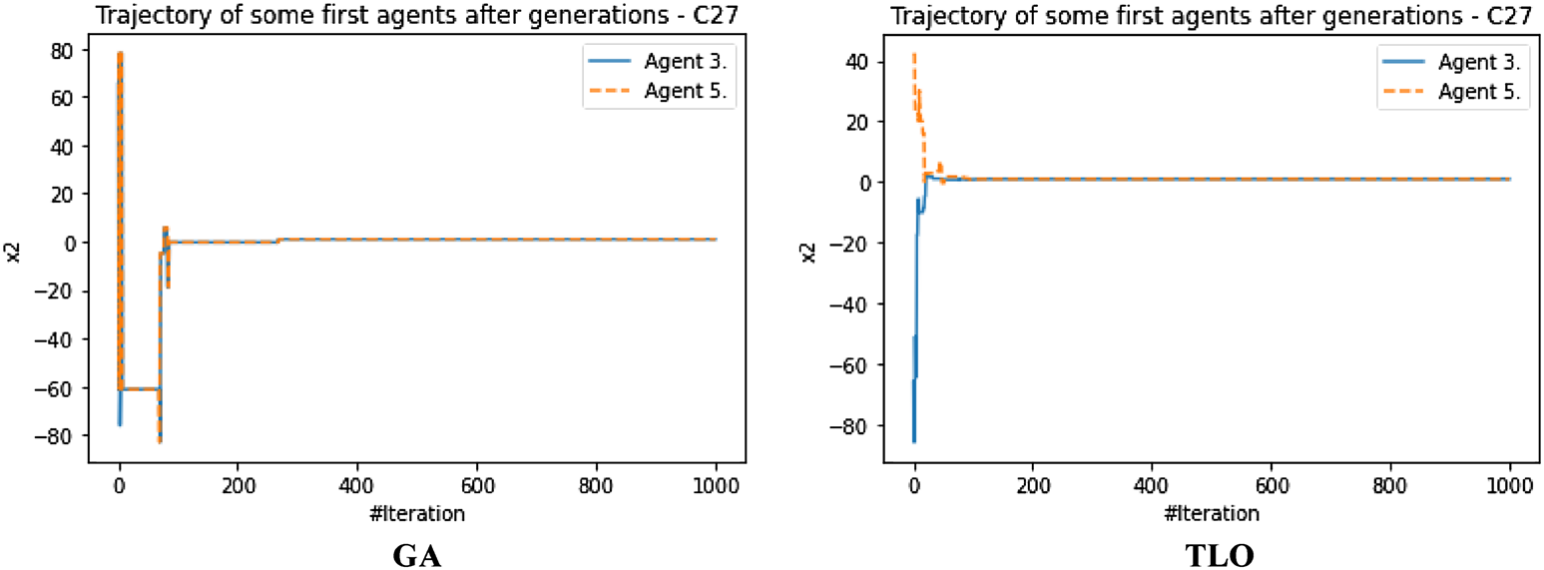
A comparative analysis of the trajectory for Shift-Rotated (SR) of Sum of Different Power (F13) for AO, EOSA, AOA, IEOSA, ArchOA, HGS, CHIO, VCS, GA, and TLO.

The performance of the new variant algorithm was also investigated on the IEEE CEC functions, and the result is listed in Table 5. The overall performance values were AO (3), AOA (3), ArchOA (3), CHIO (2), GA (0), HGS (4), IWO (2), SSA (3), TLO (2), VCS (0), WHO (2), IEOSA (3), and EOSA (0). Using five of the functions for evaluation, we found IEOSA demonstrates a competitive performance with AO, AOA, ArchOA, and SSA on these constraint benchmark functions. The HGS algorithm showed better performance when compared with the proposed algorithm on these benchmark functions. Figures 10 and 11 present the graphs for the exploration and exploitation phases of the algorithms and the trajectory of the optimization process for some selected solutions/agents. The result obtained and charted on the graphs for the exploration and exploitation process showed that IEOSA could explore solutions globally, while its local search also performs well. This good performance is repeated in the cases of AOA, AO, ArchOA, CHIO, GA, and HGS, but failed in VCS. Similarly, we see an impressive chart for IEOSA and EOSA in the trajectory of the optimization process for the two selected agents 3 and 5. This effective performance in the search for the optimization process outperformed what is seen for GA, VCS, CHIO, and ArchOA. Considering these reports on the performance of the new variant algorithm on these benchmark functions, we proceed to report the application of the algorithm.

**Table 5:** Performance evaluation of AO, AOA, ArchOA, CHIO, GA, HGS, IWO, SSA, TLO, VCS, WHO, EOSA, and IEOSA using five (5) IEEE CEC constraint benchmark functions.

Function	Metric	AO	AOA	ArchOA	CHIO	GA	HGS	IWO	SSA	TLO	VCS	WHO	IEOSA	EOSA
CEC03	Best	3.36E−02	0.00E+00	2.84E−100	3.06E+03	7.48E+03	0.00E+00	1.34E+04	3.83E−40	3.09E−246	3.08E−04	2.54E−02	0.00E+00	6.54E+03
	Mean	1.48E+04	0.00E+00	1.60E+04	1.46E+04	5.02E+05	6.46E+01	1.34E+04	1.04E+00	5.23E+03	1.64E−01	1.58E+02	5.91E+02	3.38E+05
	Std	2.44E+05	0.00E+00	2.68E+05	1.27E+05	1.08E+06	1.16E+03	3.64E−12	2.18E+01	1.59E+05	2.25E+00	1.09E+03	1.17E+04	4.95E+05
	Worst	7.66E+06	0.00E+00	5.92E+06	1.80E+06	2.87E+06	2.69E+04	1.34E+04	4.87E+02	5.02E+06	6.83E+01	1.91E+04	3.57E+05	2.05E+06
	Median	4.38E+03	0.00E+00	4.40E−34	3.06E+03	7.48E+03	0.00E+00	1.34E+04	6.00E−23	6.08E−120	4.05E−02	1.08E−01	0.00E+00	2.05E+04
	Deviation	− 3.36E−02	0.00E+00	− 2.84E−100	− 3.06E+03	− 7.48E+03	0.00E+00	− 1.34E+04	− 3.83E−40	− 3.09E−246	− 3.08E−04	− 2.54E−02	0.00E+00	− 6.54E+03
CEC06	Best	0.00E+00	0.00E+00	0.00E+00	0.00E+00	3.12E−04	0.00E+00	0.00E+00	0.00E+00	0.00E+00	4.48E−03	0.00E+00	0.00E+00	1.91E+00
	Mean	9.87E−04	0.00E+00	0.00E+00	1.13E−02	1.02E−02	0.00E+00	3.60E−04	7.40E−04	2.00E−06	2.62E−01	0.00E+00	0.00E+00	2.28E+00
	Std	2.20E−02	0.00E+00	0.00E+00	1.41E−01	9.99E−02	0.00E+00	1.14E−02	2.34E−02	6.32E−05	3.10E−01	0.00E+00	0.00E+00	6.18E−01
	Worst	4.93E−01	0.00E+00	0.00E+00	2.25E+00	2.97E+00	0.00E+00	3.60E−01	7.40E−01	2.00E−03	2.90E+00	0.00E+00	0.00E+00	3.86E+00
	Median	0.00E+00	0.00E+00	0.00E+00	0.00E+00	3.83E−03	0.00E+00	0.00E+00	0.00E+00	0.00E+00	2.40E−01	0.00E+00	0.00E+00	1.91E+00
	Deviation	0.00E+00	0.00E+00	0.00E+00	0.00E+00	− 3.12E−04	0.00E+00	0.00E+00	0.00E+00	0.00E+00	− 4.48E−03	0.00E+00	0.00E+00	− 1.91E+00
CEC07	Best	0.00E+00	0.00E+00	0.00E+00	6.05E−01	4.56E−02	0.00E+00	1.35E+00	7.11E−15	1.23E−02	3.17E−06	2.44E−05	3.34E+00	1.19E+00
	Mean	7.78E−03	0.00E+00	1.24E−01	6.99E−01	9.17E−02	4.03E−03	1.35E+00	2.54E−07	4.02E−02	1.29E−03	7.74E−03	3.34E+00	1.43E+00
	Std	5.92E−02	0.00E+00	2.30E−01	1.97E−01	1.16E−01	5.11E−02	6.54E−03	7.87E−06	9.28E−02	2.38E−02	4.78E−02	0.00E+00	1.61E−01
	Worst	9.70E−01	0.00E+00	1.51E+00	1.70E+00	1.82E+00	1.05E+00	1.50E+00	2.49E−04	1.29E+00	7.54E−01	8.94E−01	3.34E+00	1.60E+00
	Median	0.00E+00	0.00E+00	0.00E+00	6.73E−01	5.32E−02	0.00E+00	1.35E+00	7.11E−15	1.25E−02	1.98E−04	4.08E−05	3.34E+00	1.56E+00
	Deviation	0.00E+00	0.00E+00	0.00E+00	− 6.05E−01	− 4.56E−02	0.00E+00	− 1.35E+00	− 7.11E−15	− 1.23E−02	− 3.17E−06	− 2.44E−05	− 3.34E+00	− 1.19E+00
CEC10	Best	0.00E+00	0.00E+00	0.00E+00	0.00E+00	2.72E−02	0.00E+00	0.00E+00	0.00E+00	0.00E+00	5.59E−01	0.00E+00	0.00E+00	1.52E+00
	Mean	2.76E−03	0.00E+00	1.46E−04	7.05E−03	1.39E−01	0.00E+00	0.00E+00	2.51E−03	8.94E−05	2.35E−01	0.00E+00	0.00E+00	2.15E+00
	Std	4.15E−02	0.00E+00	4.62E−03	8.81E−02	2.82E−01	0.00E+00	0.00E+00	6.75E−02	2.83E−03	8.39E−01	0.00E+00	0.00E+00	9.98E−01
	Worst	9.52E−01	0.00E+00	1.46E−01	1.40E+00	4.77E+00	0.00E+00	0.00E+00	2.10E+00	8.94E−02	5.59E−01	0.00E+00	0.00E+00	4.61E+00
	Median	0.00E+00	0.00E+00	0.00E+00	0.00E+00	5.33E−02	0.00E+00	0.00E+00	0.00E+00	0.00E+00	− 9.05E−02	0.00E+00	0.00E+00	1.82E+00
	Deviation	0.00E+00	0.00E+00	0.00E+00	0.00E+00	− 2.72E−02	0.00E+00	0.00E+00	0.00E+00	0.00E+00	− 1.24E+00	0.00E+00	0.00E+00	− 1.52E+00
CEC12	Best	3.68E−02	5.00E−01	1.10E−01	1.55E+02	6.59E−01	1.67E−01	3.02E+03	1.21E−03	7.92E−02	2.99E−01	1.85E−01	5.00E−01	2.80E+03
	Mean	3.75E+01	5.00E−01	8.17E+00	2.46E+02	8.24E+00	2.68E+00	3.02E+03	3.18E−03	7.12E+00	3.35E−01	1.51E+00	1.07E+01	2.81E+03
	Std	1.90E+02	0.00E+00	1.07E+02	6.87E+02	4.36E+01	7.58E+01	5.49E+01	2.41E−02	1.42E+02	8.17E−01	3.65E+01	2.85E+02	1.35E+01
	Worst	2.92E+03	5.00E−01	2.92E+03	8.46E+03	1.15E+03	2.40E+03	3.60E+03	3.96E−01	4.30E+03	2.61E+01	1.15E+03	8.96E+03	2.87E+03
	Median	5.69E−01	5.00E−01	1.27E−01	1.55E+02	6.83E−01	1.67E−01	3.02E+03	1.21E−03	1.31E−01	2.99E−01	2.18E−01	5.00E−01	2.80E+03
	Deviation	− 3.68E−02	− 5.00E−01	− 1.10E−01	− 1.55E+02	− 6.59E−01	− 1.67E−01	− 3.02E+03	− 1.21E−03	− 7.92E−02	− 2.99E−01	− 1.85E−01	− 5.00E−01	− 2.80E+03
C16	Best	1.60E+03	1.60E+03	1.60E+03	1.60E+03	1.60E+03	1.60E+03	1.60E+03	1.60E+03	1.60E+03	1.60E+03	1.60E+03	1.60E+03	1.60E+03
	Mean	1.60E+03	1.60E+03	1.60E+03	1.60E+03	1.60E+03	1.60E+03	1.60E+03	1.60E+03	1.60E+03	1.60E+03	1.60E+03	1.60E+03	1.60E+03
	Std	6.14E−02	2.12E−02	1.49E−01	1.36E−01	7.79E−02	7.13E−02	4.55E−13	7.70E−02	8.99E−02	0.00E+00	6.22E−03	1.93E−05	0.00E+00
	Worst	1.60E+03	1.60E+03	1.60E+03	1.60E+03	1.60E+03	1.60E+03	1.60E+03	1.60E+03	1.60E+03	1.60E+03	1.60E+03	1.60E+03	1.60E+03
	Median	1.60E+03	1.60E+03	1.60E+03	1.60E+03	1.60E+03	1.60E+03	1.60E+03	1.60E+03	1.60E+03	1.60E+03	1.60E+03	1.60E+03	1.60E+03
	Deviation	− 1.60E+03	− 1.60E+03	− 1.60E+03	− 1.60E+03	− 1.60E+03	− 1.60E+03	− 1.60E+03	− 1.60E+03	− 1.60E+03	− 1.60E+03	− 1.60E+03	− 1.60E+03	− 1.60E+03
Total		3	3	3	2	0	4	2	3	2	0	2	3	0

**Figure 12:**
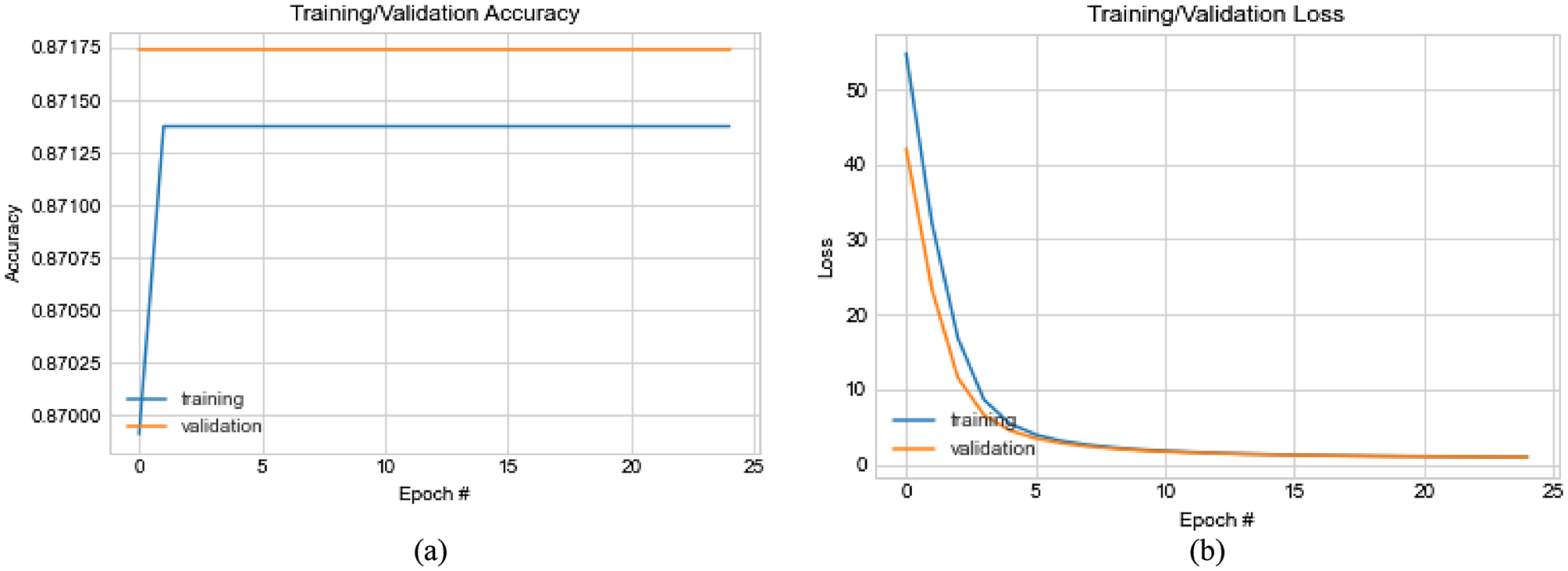
A plot of the values obtained for accuracy for training and evaluation (**a**), and loss function values for training and validation for the CNN model used for feature extraction (**b**).

The optimization problem investigated in this study is that the algorithm seeks to find the minimum number of features for each sample in a dataset required to achieve optimal classification accuracy. The aim is to optimize the feature extraction and selection process and reduce undue bottleneck operations on the classifier. To demonstrate this, the experimentation phase on this aspect of the study is first to train the CNN model so that features in the images suggestive of abnormalities are understood, learnt, and extracted through the convolutional-pooling layers. In Fig. 12a,b, the learning curve for the CNN model is presented, charting the accuracy and loss function values for twenty-five (25) training epochs. The result obtained for the loss values for both training and validation demonstrates that the CNN model progressively and effectively learnt the classification problem. Also, the distribution of values obtained for accuracies over the 25 epochs correspond. Considering the output of the trained model on the classification task, the features were then extracted and passed for optimization to select only discriminant features supporting the classification process.

The IEOSA optimization algorithm is applied to the feature optimization problem, and its performance is compared with some of the competing algorithms reported in previous paragraphs. Table 6 lists the performance of the CNN using all of the 25,600 features extracted by the convolutional-pooling layers. Results obtained showed that the classification of the metaheuristic-aided hybrid algorithms outperformed those of the CNN model, which bottlenecks the classifier. Whereas the value of 0.8717 was obtained for accuracy of the CNN model using all features, the performance of CNN + IEOSA demonstrates very good results when applied to select discriminant features. The value of 0.817 was obtained for precision when all features extracted were passed to the classifier. However, the result of the hybrid process using the optimization algorithm showed better recall and F1-score values of 1.0 and 0.60 for CNN + IEOSA, and CNN + EOSA, respectively. This performance of IEOSA as applied to this optimization problem showed how effective it is in addressing the challenge of feature selection and reduction in medical images. This result implies that noisy features could impair the performance of classifiers, thereby affecting classification reports.

**Table 6:** Performance evaluation of the classification accuracy, precision, recall and F1-score for the CNN model, using all extracted features and softmax function for classification.

Model	Accuracy	Precision	Recall	F1-score	Features
CNN	0.8717	0.8717	0.8717	0.8717	25,600

The number of features resulting from using the IEOSA algorithm for optimization purposes is shown in Table 7. This result showed that, compared with the 25,600 features extracted for each image, only reduced and selected features were required to achieve the classification operation. The relevance of this result is corroborated by the corresponding results obtained for the hybrid algorithms during the classification operation. To widen the scope of the classification process, we investigated the effect of this feature selection on some other popular classifiers, in addition to the softmax function applied for classification in the CNN model. The support vector machine (SVM), decision tree, Naïve Bayes, and k-nearest neighbor (KNN) classifiers were investigated. The results obtained showed that the optimization process for IEOSA in the feature selection problem outperformed all related hybrid algorithms in some of the classifiers. For example, the minimization task achieved by IEOSA on the number of features selected showed that 3,200 out of the total 25,600 features were used for the classification process. This is compared with EOSA, which selected 3367 features for the classification task. Also, we found that, for F1-score and recall, respectively, IEOSA returned 0.51 and 1.0 for SVM, 0.48 and 0.76 for Naïve Bayes, 0.58 and 0.72 for KNN, and 0.51 and 1.0 for decision tree classifiers. This compared with what was returned for EOSA, which are 0.54 and 1.0, 0.50 and 0.75, 0.60 and 0.85, 0.54 and 1.0, respectively for SVM, decision tree, Naïve Bayes, and KNN, under the metrics F1-score and recall.

**Table 7:** Comparative analysis of the number of features selected and classification accuracy, precision, and recall using the IEOSA and its base algorithms.

	Optimized CNN models	
	IEOSA	EOSA
**Number of features**	3200	3367
**SVM**		
F1-score	0.51	0.54
Recall	1	1
**Naive Bayes classifier**		
F1-score	0.48	0.5
Recall	0.76	0.75
**KNN classifier**		
F1-score	0.58	0.6
Recall	0.82	0.85
**Decision tree classifier**		
F1-score	0.51	0.54
Recall	1	1

The implication of these findings is that, even when a good optimization algorithm has been applied to the task of feature reduction and selection, it is necessary to select a suitable classifier for the classification task; if not, the effect of the feature selection will be eliminated. For instance, when the same number of features are applied to SVM, decision tree, Naïve Bayes, and KNN, the values for recall obtained for SVM and decision tree classifiers were 1.0, whereas those for KNN and Naive Bayes were 0.85 and 0.76, respectively. This shows that the classifier’s performance must not be chosen, such that the classification of selected features is ineffective.

The findings of the result of the experiment in this study showed that not all features extracted during the convolutional process are relevant to the classification operation. This is because such a collection of features often contains subsets of discriminant, noisy, and low-effect features. It is, therefore, very important to eliminate non-suggestive features before passing the detected features to the classifier. Therefore, this finding indicates that the performance of deep learning models, CNN specifically, can be further improved when detected features are subjected to an effective selection process for feature reduction operation. The outcome of such selection leading to reduction is that classifiers are not overwhelmed with an unnecessary number of features per sample during the classification process. An overwhelmed classifier will at some point misclassify some samples, leading to increased false positives or false negative rates. Literature has confirmed that the adverse impact of patients obtaining false positive or false negative results has raised the number of casualties recorded for the disease. When this concept of feature selection to achieve reduction is applied, the classification accuracy of the classifiers in classing abnormalities, benign cases, and normal cases will improve. In turn, the computational solution’s confidence and acceptability level will also increase.

Another interesting finding from the study is the relevance of optimization methods using metaheuristic algorithms to address the difficult problem of selecting a subset of features to sufficient for the classification operation. Most of the optimization algorithms applied showed good performance in reducing the number of selected features. Meanwhile, the use of IEOSA and its performance on this classification problem showed that the optimization algorithm to be chosen for this task must support the optimization problem.

We note that the ability of the proposed IEOSA algorithm to reduce the number of features from 25,600 extracted by the CNN models to only 3,200, which were eventually used for classification, is a very significant optimization performance. This further corroborates the results obtained when the benchmark functions were applied for evaluating IEOSA with similar algorithms. The emphasis of these findings is that a bottlenecked classifier will be overwhelmed, leading to reduced classification accuracy and impaired performance in all other cases. Interestingly, this algorithm does not only lead to improved classification accuracy. The finding from the research also confirms the relevance of using metaheuristic algorithms for addressing the difficult problem of feature selection. This study has extended research in feature selection beyond the classical use of optimization algorithms for the selection of features in text-based datasets, since image-level datasets are considered in the study. This assertion is based on the fact that features extracted from digital medical images are characterized by a very high dimensional number of features. The approach and algorithm proposed in this study have been able to reduce these high-dimensional features to only significant and discriminant features required for classification purposes. As a future work in this direction, we motivate to investigate very difficult digital images, such as histopathology images, which are known to come with three channels of colour and are stained by the pathologist. This is recommended, seeing that the use of IEOSA with grey images demonstrated outstanding performance, and it should be considered suitable to address a more difficult feature selection problem as described here.

## Conclusion

This study presents a new variant of the EOSA algorithm based on the population immunity concept to achieve the proposed IEOSA method. The new variant was improved with the chaotic theory technique for the initialization of the initial solutions. Moreover, the study aimed to select an optimal subset and number of features from those extracted during the convolutional-pooling operations. The purpose of this approach is to allow for only the most relevant features, possessing discriminant properties which can support the classification operation. This expected outcome is to improve the classification accuracy, which should translate to reduced false positive or false negative rates. Using a CNN architecture applied in our recent study, the relevant features were selected using the new variant algorithm, that is the IEOSA. The results showed that the optimization process’s performance in eliminating non-relevant features yielded better performance than when all features were applied to the classifier. Therefore, the contribution of this study emphasizes the need for supporting the feature classification process in medical images with optimization algorithms to reduce the number of features per image used for the classification process. Meanwhile, the outcome of this study opens research frontiers to designing state-of-the-art classifiers capable of self-optimizing features so that external mechanisms will not be required to support their operations.

In the future, the IESOA can further be enhanced by improving its search strategy and using more effective deep learning models. In addition, further validation and evaluation of the IESOA’s superior performance can be made by comparing it with other recently implemented state-of-the-art metaheuristics algorithms such as the Farmland Fertility (FF) algorithm, African Vultures Optimization Algorithm (AVOA), and Artificial Gorilla Troops Optimizer (GTO). Finally, considering the inherent practical performance potential of the new method, the IESOA can be applied to solve other real-world application problems.

## Data Availability

All data generated or analysed during this study are included in this published article.
